# Alanine scanning of the yeast killer toxin K2 reveals key residues for activity, gain-of-function variants, and supports prediction of precursor processing and 3D structure

**DOI:** 10.1016/j.yjsbx.2025.100142

**Published:** 2025-12-18

**Authors:** Rianne C. Prins, Tycho Marinus, Eyal Dafni, Iftach Yacoby, Sonja Billerbeck

**Affiliations:** a Molecular Microbiology, Groningen Biomolecular Sciences and Biotechnology institute, University of Groningen, Groningen 9747 AG, the Netherlands; b Chemical Biology 1, Groningen Biomolecular Sciences and Biotechnology institute, University of Groningen, Groningen 9747 AG, the Netherlands; c School of Plant Sciences and Food Security, The George S. Wise Faculty of Life Sciences, Tel Aviv University, Tel Aviv 6997801, Israel; d Department of Bioengineering, Imperial College London, South Kensington Campus, London SW7 2AZ, UK

**Keywords:** Yeast killer toxin, Alanine scanning, K2, Oligo pools, Sequence-function map, Gain-of-function, Precursor processing, Structure prediction, DUF5341

## Abstract

Yeast killer toxins (YKTs) are antimicrobial proteins secreted by yeast with potential applications ranging from food preservation to therapeutic agents in human health. However, the practical use of many YKTs is limited by specific pH requirements, low temperature stability, low production yields, and narrow target specificity. While protein engineering could potentially overcome these challenges, progress is hindered by a lack of detailed knowledge about sequence-function relationships and structural data for these often multi-step processed proteins. In this study, we focused on the YKT K2, encoded by the M2 satellite dsRNA in *Saccharomyces cerevisiae*. Using alanine scanning mutagenesis of the full open reading frame and structure predictions combined with molecular dynamics simulations, we generated a comprehensive sequence-structure–function map, refined the model for the proteolytic processing of the K2 precursor, and predicted the mature toxin structure. Our findings also demonstrate that K2 can be engineered toward enhanced toxicity and altered target specificity through single-site mutations. Furthermore, we identified structural homology between K2 and other killer toxins, including the SMK toxin from the yeast *Millerozyma farinosa*. Our cost-effective workflow provides a platform to broadly map YKT sequence-structure–function relationships.

## Highlights

• Mutational scanning indicates more processing sites in K2 than initially recognized.
• Insights into precursor processing help to predict the mature toxin structure.
• Single mutations can enhance killer toxin function and change target specificity.
• Yeast killer toxins are part of structural families despite low sequence identity.

## Graphical abstract

**Figure:**
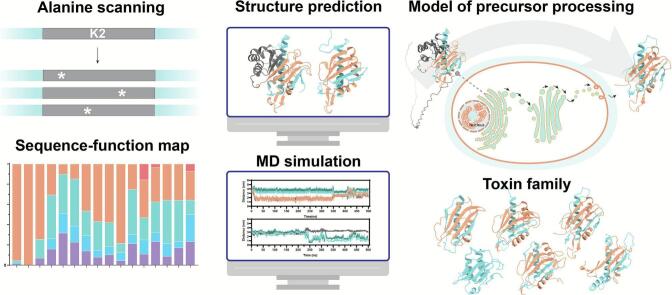

## Introduction

Many yeasts secrete antifungal proteins that are lethal to sensitive strains, providing a competitive advantage in ecological niches and shaping microbial communities (Crabtree, 2023, Mannazzu, 2019, Boynton, 2019). With a growing threat of antifungal resistance and a need for novel antifungal agents (van Rhijn, 2024), these functionally diverse yeast killer toxins (YKTs) hold potential for various applications, from biocontrol agents for crops and food to therapeutic agents for human health (Mannazzu, 2019, Billerbeck, 2024).

However, there are intrinsic limitations that need to be overcome. The natural producers of killer toxins often thrive in ambient temperatures and acidic environments, rendering many killer toxins dependent on low pH and showing low thermal stability, with often low production yields (Billerbeck, 2024). Since these toxins are ribosomally synthesized proteins, their encoding genes are potentially amenable to engineering toward overcoming these limitations (Gillor, 2005). In addition, it would be of interest to engineer target ranges of these toxins toward narrow- or broad-spectrum applications (Billerbeck, 2024).

**Fig. 1:**
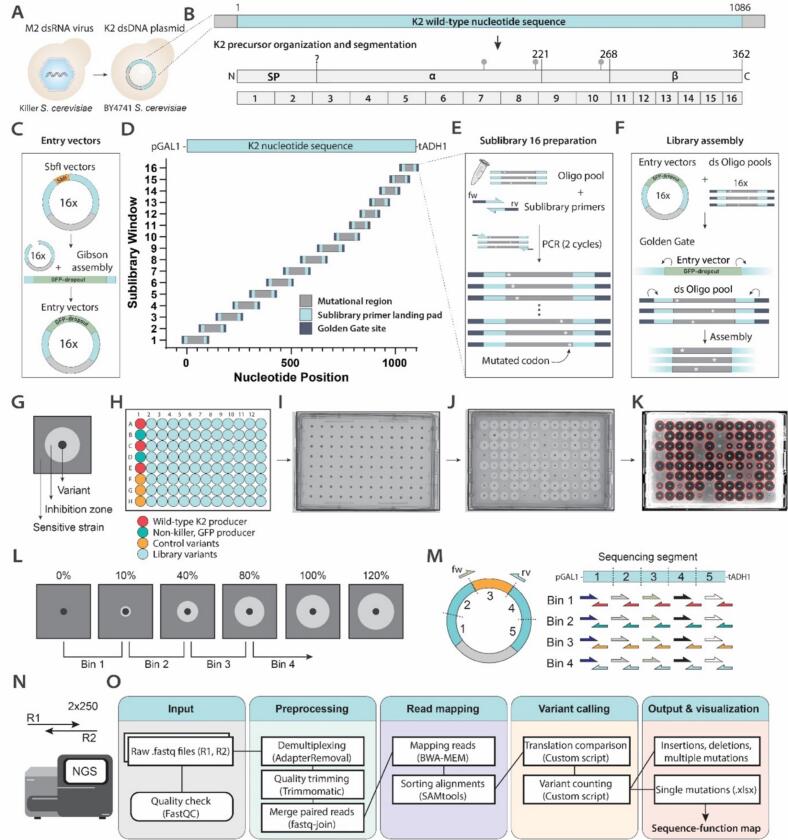
Overview of alanine-scanning library creation, variant screening, and next-generation sequencing. (A) The K2 killer phenotype is naturally conveyed by the presence of the M2 dsRNA satellite in *S. cerevisiae*. Within this study, the sequence encoding K2 was expressed from a pRS423-type dsDNA plasmid. (B) The product of the ORF is the K2 precursor, which consists of 362 residues, reportedly organized in a signal peptide (SP) and the α and β domains that form the mature toxin. The exact signal peptidase cleavage site is unknown. Two potential canonical dibasic (KR) Kex2p protease cleavage sites are present at residues 220/221 and 267/268. Three potential N-glycosylation sites are present at N177, N214 and N261. The ORF was divided into 16 segments to facilitate the cloning of sublibraries using pooled mutant oligonucleotides (**Supplementary Table S1** and **S14-S16**). (C) Each of the 16 segments was previously replaced by an SbfI restriction site (Prins and Billerbeck, 2024), which was used here to insert a GFP-dropout module. (D) Each segment sequence was encoded on oligonucleotides in which alanine substitutions were introduced. The mutant oligonucleotides were ordered as 16 sublibrary pools. (E) The single-stranded oligonucleotides were double-stranded and type-II restriction sites were added. (F) Within a Golden Gate reaction, the GFP-dropout modules were replaced by the respective mutant oligonucleotide pools, introducing the alanine conversions into the wild-type K2 sequence. (G) The resulting variants were assessed for formation of a zone of inhibition on a lawn of a sensitive indicator strain, indicating secretion of active toxin. The size of the halo is related to the activity level of the toxin. (H) *S. cerevisiae* transformants were arrayed into 96-well microtiter plates. In each plate, the first column was dedicated to controls: Three replicates of wild-type K2 producers, two replicates of a non-killer that produces GFP (convenient for determining plate orientation), and three previously constructed control variants with varying expected halo sizes. (I) The cultures were first pinned in duplicate onto solid media, under conditions inducing protein expression, to create the source plates. (J) From the solid source plates, the colonies were replicated onto an assay plate containing an embedded sensitive strain and halo sizes (zones of inhibition) were imaged after two days. (K) The areas of the zones of inhibition were quantified using image-processing software (Dafni, E., et al., 2019), adjusted for this purpose. The library was screened in duplicate and the displayed images are representative of the results. (L) The different halo area sizes were compared to the wild-type zone of inhibition of the K2 toxin (set to 100 %). The variants were divided into four bins based on the area of the halo: Bin 1 (0–10 %), bin 2 (10–40 %), bin 3 (40–80 %) and bin 4 (>80 %). (M) The K2 sequence was divided into five segments for next-generation amplicon sequencing. Amplicons were generated using primer combinations specific for each segment/bin combination that add barcodes and partial Illumina adapters (**Supplementary Table S4**). (N) Samples were sequenced using a NGS Illumina platform, yielding 250 bp paired-end reads that cover the entire mutated region. (O) Reads were assessed for quality, demultiplexed based on the bin/segment specific barcodes, mapped to the template plasmid, and counts of single, intended, amino acid conversions within each sample were determined using a custom script.

K2 is a killer toxin encoded on a satellite dsRNA (M2) in *Saccharomyces cerevisiae* (Fig. 1A) (Dignard, 1991)*.* The K2 toxin is produced by commercial wine yeast strains (Billerbeck, 2024, Zhong, 2024) and is most active at a temperature of 20–25 °C and under acidic conditions − it loses activity completely above pH 5.5 (Lukša, 2016). It is active against several yeasts, including sensitive *S. cerevisiae* strains as well as clinically relevant isolates of *Nakaseomyces glabratus* (previously named *Candida glabrata*), which can have high levels of resistance to current clinical antifungal therapeutics (Fredericks, 2021). Therefore, the K2 toxin serves as an ideal model for exploring toxin bioengineering opportunities.

The K2 toxin precursor consists of 362 amino acids (Fig. 1B). The N-terminal signal peptide directs the downstream amino acid sequence into the secretory pathway and simultaneously functions as the immunity factor that prevents self-killing (Prins and Billerbeck, 2024). Within the early secretory pathway, disulfide bridge formation and putative glycosylation take place (Dignard, 1991). Within the late Golgi, proteolytic processing occurs by the endopeptidases Kex1p and Kex2p which finally yields the α and β subunits that form the mature secreted heterodimeric K2 toxin (Dignard, 1991). Toxin molecules subsequently diffuse into the environment, dock onto β-1,6-glucan within the yeast cell wall of target cells (Lukša, 2015), likely interact with the plasma membrane receptor Kre1p (Novotna, 2004) and then act as ionophores to induce membrane permeability (Schmitt and Breinig, 2006, Orentaite, 2016) – a process in which the rather hydrophobic α subunit seems to play a critical role (Prins and Billerbeck, 2024).

The exact molecular mechanism by which the K2 toxin kills cells is however not completely understood. The lack of efficient protocols to purify the secreted K2 toxin from the culture media has hindered structural studies. Previous studies have investigated the importance of only a limited number of residues by site-directed mutagenesis, such as two putative protease processing sites (Fig. 1B) (Dignard, 1991). Without precise data on precursor processing sites, the prediction of the mature toxin subunits and the associated three-dimensional (3D) protein structure is challenging. Gaining insights into sequence-function relationships and the mature toxin structure is essential for advancing our molecular understanding of YKTs and facilitating rational design in engineering strategies.

To gain insights into the sequence-function relationships of the K2 toxin, we performed a systematic alanine scanning mutagenesis of the full K2 open reading frame (ORF). Here, each substitution into alanine examines the contribution of individual side chains to the functionality of the toxin. Recent advancements in protein structure prediction further contributed to the generation of precursor and mature toxin structure predictions (Evans, 2021, Jumper, 2021, Mirdita, 2022), which were further refined by molecular dynamics simulations. Lastly, we performed sequence-based searches for homologous sequences to identify potential conserved residues.

This workflow yielded a sequence-function map that identified critical residues for toxin activity and yielded variants with enhanced function and altered species-specificity, showing the engineering potential of K2. Further, it enabled us to create a new model for K2 precursor processing and to predict the mature K2 toxin structure. We thereby identified structural similarities with the mature SMK toxin from the yeast *Millerozyma farinosa*, for which a crystal structure is available. Precursor predictions of SMK and MD simulations further supported and refined the model for K2 processing and our structure predictions. Eventually, this allowed us to expand our sequence-function map to a sequence-structure–function map. An extended analysis also identified a putative family of YKTs with structural similarities: KP4, SMK, K2, K1, K66, K45 and KHS1, as well as other proteins containing the domain of unknown function 5341 (DUF5341) which is present in some toxins.

## Results

### Creation of a sequence-function map identifies critical residues and yields gain-of-function variants with higher toxicity and altered targeting specificity

The alanine scanning mutagenesis library was created using a plasmid-based system with a galactose-inducible promoter (Fig. 1) and by using our previously described segmentation and oligo pool-based Golden Gate cloning protocol (Kuiper, B.P., et al., 2022, Valero, 2025). The alanine scan involved all codons of the K2 ORF besides the stop codon (362 codons) and was cloned as 16 sublibrary segments **(**Fig. 1B**,** **Supplementary Table S1)**. Native alanine residues were converted into glycine.

The library was cloned in *Escherichia coli* and then used to transform *S. cerevisiae* BY4741. Individual colonies were arrayed into a 96-well format and subjected to a halo assay to assess toxin activity (Fig. 1**G-J**). This coupled toxin activity to a screenable phenotype, since the size of the formed zone of inhibition was related to toxin functionality (Prins and Billerbeck, 2025). The assay provides a stringent test for function: The toxin needs to be efficiently transcribed, translated, processed, folded, secreted, and needs to be stable and to diffuse in the agar media and interact properly with target cells. In total, 1228 yeast colonies were screened, providing a theoretical probability of 0.96 for library completeness (**Supplementary Table S2 and S3**).

The variants created a variety of sizes of zones of inhibition (halos) (Fig. 1J), which were quantified using a modified version of the image-processing software CFQuant (Fig. 1K) (Dafni, E., et al., 2019). We determined a confidence interval for wild-type toxin activity between 80–120 % of the average wild-type halo size (see **Methods**) and then divided the variant colonies into 4 activity bins from loss-of-function to wild-type activity (Fig. 1L). We observed that 13.2 % of all the screened colonies showed loss-of-function (bin 1, 0–10 % of wild-type halo size), 9.7 % showed a major negative effect (bin 2, 10–40 %), 26.1 % showed a moderate negative effect (bin 3, 40–80 %), and 49.8 % of the colonies retained wild-type level activity (bin 4, >80 %) (**Supplementary Fig. S1,** **Supplementary Table S5**). Interestingly, some colonies (1.3 %) created increased halo sizes (>120 %) (**Supplementary Fig. S1**).

To create genotype-phenotype links, we pooled the colonies per bin and sequenced them by NGS using paired-end reads and a tiling approach (Fig. 1**L-N**, **Supplementary Table S4**) (Valero, 2025). The few variants with > 120 % activity were in addition individually sequenced by Sanger sequencing. The distribution of read counts of single, intended mutations followed a Gaussian distribution with 50 % of positions showing between 243 and 684 variant read counts, eleven variants did not yield any reads and eleven variants were significantly overrepresented (**Supplementary Fig. S2**).

K2 variants were assigned functional scores from 1 (loss of function, bin 1) to 4 (wild-type function, bin 4) based on their bin distribution. Variants spanning two adjacent bins were given averaged scores (1.5, 2.5, or 3.5), which was the case for three, 16, and 40 variants, respectively. In this way, we were able to determine functional scores for 340 out of the 362 positions (93.9 %), rendering the library near-complete (**Supplementary Table S6 and S7**). A score of 0 was given to variants where data did not meet our set quality thresholds: 22 positions (6.1 %) were excluded due to insufficient read counts (12 cases) or reads significantly distributed across non-adjacent bins (ten cases). The latter likely reflected mixed colonies, dual-plasmid populations, or contamination during handling.

**Fig. 2:**
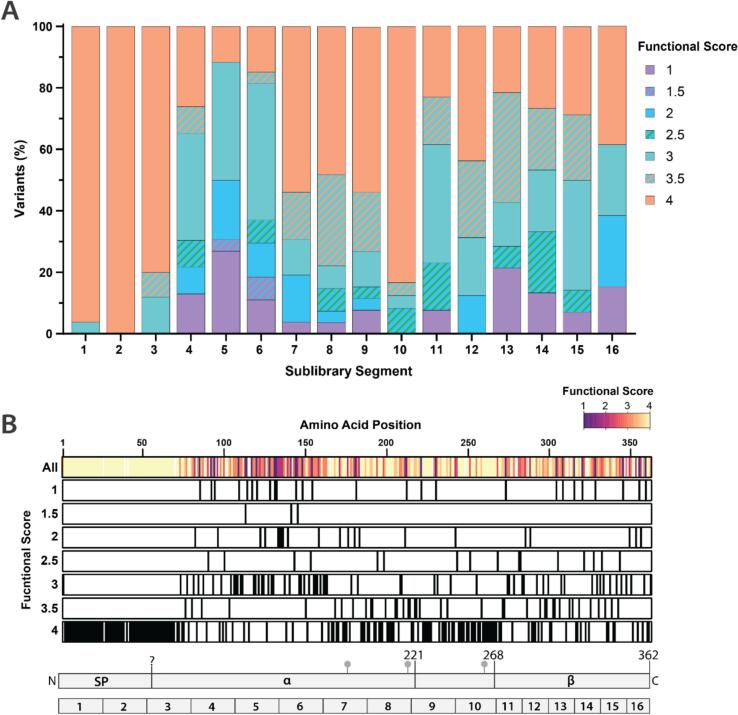
**Variant functional scores distribution along the K2 ORF.** (**A**) Distribution of the functional scores per sublibrary segment of the K2 ORF (**Supplementary Table S1**). Per segment, the percentage of all positions within that segment with a given functional score is indicated. (**B**) Functional score distribution displayed along the K2 ORF for individual positions. The 16 sublibrary segments are displayed below for reference.

From the resulting data we created a sequence-function map, here presented by the global distribution of functional scores along the K2 ORF (Fig. 2, Fig. 2) or by a detailed per-residue function map (**Supplementary Fig. S3**).

Overall, 50.0 % of alanine (or alanine to glycine) conversions resulted in wild-type toxin activity (score 4) and 7.7 % (26 residues) lost toxicity (score 1) (**Supplementary Fig. S3,** **Supplementary Table S6 and S7**), indicating that the latter side chains performed a highly important function for toxicity. Alanine substitutions are considered relatively mildly disruptive compared to some other amino acids, thus our results were consistent with this type of scan mainly identifying key residues for function (Gray, 2017).

Many of the 26 critical residues were hydrophobic and aromatic (**Supplementary Table S6**): four of the five tryptophan (W) and six of the 26 tyrosine (Y) variants lost function when mutated into alanine. Further, two glycine residues (G144, G345) were critical, and one alanine residue (A131) could not be converted into glycine without loss of function. The six critical cysteine residues were all located within the precursor region predicted to be part of the mature toxin (**Supplementary Table S6**), while one of the three non-critical cysteines was also present in this region and the remaining two were found in the predicted signal peptide of K2 **(**Fig. 1B**,** **Supplementary Table S6)**. Overall, this suggested that three critical disulfide bridges were formed within the mature protein. The three predicted *N*-glycosylation sites (N177, N214 and N261, Fig. 1B) were not critical for function.

**Fig. 3:**
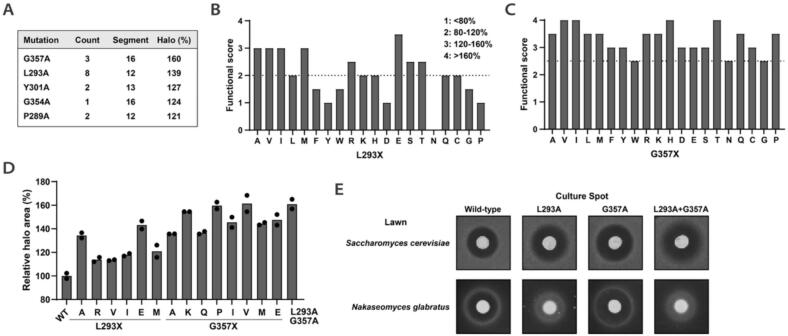
**Gain of function variants and variants with altered target specificity**. (**A**) 16 colonies with increased halo sizes were Sanger sequenced, and several mutations associated with gain of function were identified. For the relative average halo area size, the wild-type halo was set to 100 %. (**B**) The determined functional scores of the saturation mutagenesis library of position 293 are displayed. The horizontal line indicates the wild-type. Bin borders specific for figure panels B and C are displayed in the right top corner. (**C**) The determined functional scores of the saturation mutagenesis library of position 357 are displayed. The horizontal line indicates the wild-type **(Supplementary Note 1)**. (**D**) Plasmids were extracted from library variants, transformed in *E. coli*, purified from *E. coli* and reintroduced into *S. cerevisiae.* The resulting transformants were screened in duplicates for production of a zone of inhibition, of which the area was quantified after 2 days of growth (displayed relative to the average of the wild-type, which was set to 100 %) (**Supplementary Fig. S5**). WT; plasmid encoding wild-type K2. Dots indicate the individual replicate values, while the bar indicates the mean. (**E**) The OD_600_ of *S. cerevisiae* duplicate overnight cultures − expressing either wild-type K2, K2 with an L293A or G357A mutation or L293A + G357A − was normalized and 5 µL was spotted on top of either a *S. cerevisiae* pbs2Δ::KanMX4 or *N. glabratus* indicator lawn. Zones of inhibition were imaged after two days of growth.

Next to the critical residues, we investigated the gain-of-function variants that showed increased halo sizes. Sanger sequencing located all of them in the predicted β subunit. Specifically, P289A, Y301A and G354A mutations resulted in relatively small increases in activity (121–127 %), while L293A and G357A mutations showed an average halo size of 139 % or 160 %, respectively **(**Fig. 3A**)**. We wondered if the large increase in activity for L293A and G357A was a specific result of a conversion into alanine, or whether other amino acid substitutions could lead to the same effect. We thus created, screened and sequenced a saturation mutagenesis library of L293 and G357 (**Supplementary Fig. S4, Supplementary Note 1,** **Supplementary Table S8**). Note that, because we were specifically interested in gain of function, we here used different bin borders than for the alanine scan; bin 1 − loss of function (<80 % halo area of wild type), bin 2 – wild-type level toxicity (80–120 %), bin 3 – gain of function (120–160 %) and bin 4 – major gain of function (>160 %).

The data revealed that multiple other amino acid substitutions resulted in increased halo sizes (Fig. 3, Fig. 3): For position 293, especially L293E, but also L293V, L293I and L293M showed increased halo sizes (Fig. 3B). Interestingly, aromatic sidechains (F, Y, W) were not well tolerated and resulted in loss of function, as well as residues that altered conformational stability of the backbone (G, P) (Fig. 3B). Position 357 was very tolerant to amino acid substitutions, with no loss-of-function mutations compared to the wild type but several substitutions leading to an increase in the halo size, especially G357V, G357I, G357H, and G357T (Fig. 3C). Overall, mutations at position G357 led to larger increases in halo size than those at position L293.

We validated the library results by extracting plasmids from selected variants, reintroducing them into *S. cerevisiae* BY4741, and assessing toxicity using a manual halo assay (Fig. 3D**, Supplementary Fig. S5**). We also constructed a double mutant containing both L293A and G357A. This variant showed an additive effect, producing a larger halo than either single mutation, suggesting that the two substitutions influence distinct aspects of toxin activity (Fig. 3, Fig. 3**, Supplementary Fig. S5**).

We further tested if these gain-of-function variants showed similar phenotypes when tested against a different target species. When tested against the human pathogen *Nakaseomyces glabratus*, the G357A mutation similarly showed an increased halo size when compared to wild-type K2, but L293A and the double mutant (L293A + G357A) showed a reduced halo size **(**Fig. 3E**)**. Several other L293 and G357 mutations that enhanced killing of *S. cerevisiae* also showed a decreased toxicity against *N. glabratus* **(Supplementary Fig. S5)**. These findings indicate that the observed effects were not due to altered secretion or diffusion but likely reflect mechanistic differences in target interaction, supporting that K2 can be engineered for enhanced potency and modified species specificity.

### Precursor processing: Evidence for a putative proregion (δ domain) and γ domain between the α and β domain processed at non-canonical processing sites

Next, we used our sequence-function map to investigate how the K2 precursor is proteolytically processed into the mature, secreted K2 toxin. This knowledge is important, since it determines the final primary sequence of the secreted K2 toxin. Many other toxins undergo extensive cleavage within the secretory pathway (Kashiwagi, 1997, Gier, 2020).

So far, the K2 precursor was thought to contain a signal peptide (preregion), followed by an α and β domain that form the mature toxin **(**Fig. 1B**)**. The exact signal peptidase cleavage site and thus the preregion/α-subunit boundary remains unknown. Two canonical dibasic (KR) Kex2p cleavage sites at residues 220/221 and 267/268 are predicted to define the α/β boundaries, although it remains unclear which of the two sites marks the β-subunit N terminus*.*

Several other dsRNA-encoded toxins, such as K1 and K28, encode additional proregions (also called δ) and γ regions that are removed by Kex1p and Kex2p in the late Golgi (Bader, 2008, Bevan, 1998). Kex2p typically recognizes and cleaves after two basic amino acids ('KR', 'RR') but can also cleave less efficiently at non-canonical ‘AR’, ‘ER’, or ‘PR’ sites (Bevan, 1998). For example, it recognizes ‘ER’ and ‘PR’ motifs within K28 and K1, respectively. Thus, the P1 residue – the residue immediately before the cleavage site in a substrate protein − needs to be an arginine (Bevan, 1998); while the P2 residue – located N-terminal from the P1 site − is flexible to some extent.

To understand K2 precursor processing, we systematically analyzed the precursor for evidence of a proregion and/or γ region by searching for additional non-canonical but essential Kex2p cleavage sites and by using mutational (in)tolerance across the precursor as an indication of functional importance. We assume that arginines may form P1 positions part of important Kex2p recognition sites when mutagenesis of the arginine into alanine results in loss of function − a rationale that has also been used before for K1 (Gier, 2020). In addition, mutationally tolerant regions may correspond to segments that are removed before secretion. We also constructed several additional variants to support this analysis.

**Fig. 4:**
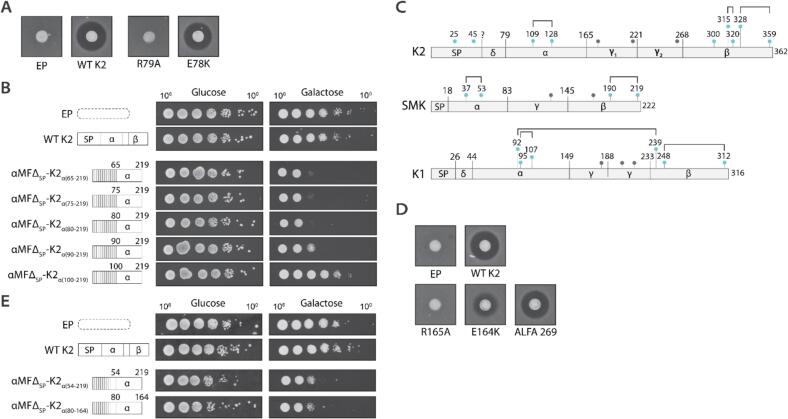
**K2 precursor organization (SP-δ-α-γ-β domains).** (**A**) The toxin activity of variants R79A and E78K was tested in a halo assay. Cell cultures, normalized for optical density, were spotted on top of agar containing a sensitive strain, and the formed zones of inhibition were imaged after two days. EP; empty plasmid, WT K2; wild-type K2. (**B**) The suicidal effect of expression of truncated K2 cytotoxic α subunit was assessed when the sequence was fused to αMF_SP_; a signal peptide of the α-mating factor. Cell cultures normalized for optical density and were diluted 1:10 (∼10^6^-10^0^ cells) and spotted onto plates with non-inducing (glucose) or inducing (galactose) conditions. Plates were incubated for three days before imaging. (**C**) Schematic overview of the new proposed K2 precursor domain organization, with the proregion (δ domain) between positions ∼ 55 and 79, the α domain from position 80–164, a γ domain spanning residues 166–266 with an internal cleavage site, and the β domain consisting of residues 269–362. Blue pins show cysteine residues with bridges indicating disulfide bonds, while grey pins locate potential *N*-glycosylation sites. (**D**) The indicated strains were spotted onto a sensitive background strain to determine toxin activity. The formed zones of inhibition were imaged after 2 days. (**E**) The suicidal phenotype of the α subunit was determined from expression of residues 54–219 or 80–164, fused to αMF_SP_. Cell cultures normalized for optical density and were diluted 1:10 (∼10^6^-10^0^ cells) and spotted onto plates with non-inducing (glucose) or inducing (galactose) conditions. Plates were incubated for three days before imaging.

We started by looking for a proregion between the signal peptide and the α subunit. Our previous work had shown that the N-terminal signal peptide of K2, which we predicted to consist of residues 1–54, was important for self-immunity, but that individual residues were not essential for K2 function (**Supplementary Note 2**). In accordance, 96 % of all variants between residues 1–54 (sublibrary segments 1 and 2) in our alanine-scanning library showed wild-type toxicity (score 4) **(**Fig. 2**,** **Supplementary Table S7**). Only the M1A mutation resulted in a decrease in halo size, consistent with previous observations (Prins and Billerbeck, 2024). Further, all analyzed residues between positions 55 and 72 resulted in wild-type-level activity, while all analyzed variants between residues 73 and 81 had at least a functional score of 3 **(**Fig. 2**, Supplementary Fig. S3,** **Supplementary Table S7**). After position 81, a stretch with more critical residues started (F82 = score 2; W85 = score 1) **(**Fig. 2**, Supplementary Fig. S3)**. In addition, we found a non-canonical (‘ER’) potential Kex2p cleavage site at position 78/79. The R79A variant showed loss of function, as expected for an important Kex2p processing site (Fig. 4A**,** this mutant was cloned separately because it was one of the missing variants that had not met our quality thresholds) while an E78K mutation, which would yield a canonical dibasic ‘KR’ site, resulted in wild-type level active toxin secretion (Fig. 4A).

Thus, we concluded that residues 1–79 could potentially encode not only the signal peptide (preregion) but also a proregion. To further substantiate this, we conducted a mutagenesis experiment to determine whether the combined preproregion was dispensable for α-subunit toxicity, as it would be removed during processing. We previously showed that expressing the α subunit alone (at the time we used residues 54–219) led to a suicidal phenotype in producer cells when replacing the K2 signal peptide with the yeast α-mating factor (αMF) signal peptide for secretion (Prins and Billerbeck, 2024). This was as expected, since the original K2 signal peptide encodes both the secretion signal but also immunity to K2, while the α-mating factor (αMF) signal peptide only directs secretion.

Using the suicidal phenotype as a readout for α-subunit functionality, we systematically truncated the predicted proregion (Fig. 4B). Deletion of residues up to position 80 had no impact on α-subunit toxicity, while deleting residues up to position 90 resulted in a reduced suicidal phenotype. Deleting residues up to position 100 did not yield a functional α subunit anymore (Fig. 4B). This confirmed that residues 1–80 were not required for α-subunit activity. We note that we observed some toxin secretion even after removal of this section, suggesting that the sequence between residues 80 and 90 can direct secretion to some extent when residues 1–79 are deleted **(Supplementary Fig. S6).** Further*,* since there are multiple potential signal peptidase cleavage sites within the signal peptide C region (Prins and Billerbeck, 2024), and since signal peptidases recognize ‘AXA’ motifs where both alanine and glycine can be found in the A position, our alanine scan was not useful in determining the exact signal peptidase cleavage site. So far, we have shown that residues up to position 80 were not required for α-subunit activity. We had shown before, with a predicted signal peptidase cleavage site after position 54, that residues 1–54 form a preregion that directs secretion and establishes immunity (Prins and Billerbeck, 2024), here, we next propose that residues 55–79 form a proregion (δ domain) that is cleaved in the secretory pathway at an essential, non-canonical Kex2p site after R79 **(**Fig. 4C**)**, yielding an α subunit starting at position 80.

Next, we investigated the C-terminal boundary of the α subunit and whether the K2 precursor, like those of K1 and K28, contains a γ subunit between the α and β subunits. Fig. 2A already revealed the functional regions of the α and β subunits, as indicated by stretches with a high percentage of functionally relevant and critical residues: Segments 4 to 6 (residues 82–162) within the α subunit, and segments 11 (residues 269–284), and 13 to 15 (residues 301–348) within the β subunit showed a low percentage of residues with wild-type activity (score 4), when compared to segments 7–10 (residues 163–268) and segment 1 to 3 (residues 1–81) **(**Fig. 2B**,** **Supplementary Table S7**). Especially segment 10 (residues 244–268) was relatively tolerant to alanine conversions compared to surrounding segments: 83 % of the variants had a functional score of 4.

This mutational tolerance suggests that a γ subunit may be present between the α and β subunits. To define its boundaries, we mapped all potential Kex2p cleavage sites and assessed their essentiality by listing each arginine residue (P1 position) and its functional score within residues 80–362: We found that variants R165A and R221A were associated with functional score 1 (consistent with previous data (Dignard, 1991) and variant R268A with a score of 2.5 (Fig. 4D**, Supplementary Fig. S3**, **Supplementary Note 3)**. As such, R165 potentially forms the P1 residue of a non-canonical ‘ER’ Kex2p cleavage site at the N terminus of the γ domain, while R221 or R268 potentially marks the C-terminal end of the γ domain. Our alanine scanning data of the P2 site of these potential Kex2p cleavage sites showed that E164A and K267A mutations were tolerated (Fig. 4D, **Supplementary Fig. S3**), which would preserve a functional Kex2p ‘AR’ recognition site (Bevan, 1998). K220A showed a minor decrease in toxicity (score 3.5), suggesting that a less efficient ‘AR’ cleavage site suffices to maintain a high activity level but that efficient cleavage is necessary for full activity, which had been shown before (Dignard, 1991). We further tested the effect of introducing a canonical KR site at position 164/165: The E164K mutation resulted in a reduced toxicity, suggesting that an ‘ER’ or ‘AR’ motif at this site provides a functional advantage compared to a canonical ‘KR’ cleavage site (Fig. 4D). Perhaps retaining the P2 residue at position 164 is beneficial for toxin function, since in the mutant E164K, the lysine would be removed by Kex1p, leaving A163 as the final C-terminal residue of the α subunit.

So far, using mutational tolerance and cleavage-site essentiality as a guide, these data indicated that the K2 α subunit consisted of residues 80–164, that the γ subunit likely consisted of residues 166–266 with an internal cleavage site at position 220–221 (resulting in regions γ_1_ and γ_2_), and that the β subunit consisted of residues 269–362 (Fig. 4C). To substantiate this hypothesis, we expressed residues 80–164 fused to the αMF signal peptide and verified that this sequence yielded a functional toxic α subunit as shown by the suicidal phenotype (Prins and Billerbeck, 2024) (Fig. 4E).

In addition, the fact that an R268A variant retained activity to some extent suggested that a short N-terminal extension of the β subunit (by region γ_2_) may be tolerated. We therefore tested the insertion of an epitope tag at this position. Insertion of the ALFA tag (Götzke, 2019) at position 269 resulted in a K2 variant that remained active (Fig. 4D).

Although mutationally more tolerant than other segments, the proposed γ region still encoded several critical residues (**Supplementary Fig. S3**), potentially by impacting proper folding and maturation of the toxin precursor. As such, the γ subunit does play a critical role in toxin function, rather than being a simple linker. Further, although three potential glycosylation sites were located within the γ subunit **(**Fig. 1B**)**, none was individually essential for function according to our alanine scan **(Supplementary Fig. S3**). Thus, even if the γ subunit is glycosylated intracellularly, its proposed cleavage suggests that the mature toxin is not *N*-glycosylated, consistent with other related toxins (Gier, 2020, Rodriguez-Cousino, N., et al., 2022) **(Supplementary Note 4).**

Our novel proposed K2 ORF gene organization, including a γ subunit, resembles that of the K1 and SMK toxins **(**Fig. 4C**).** In particular, the presence of a proregion and γ subunit resembles that of K1, which also harbors an internal γ-subunit cleavage site (Zhu, 1992). In K1, this R188A mutation showed strongly reduced but not a complete loss of function, while this cleavage site in K2 appears critical for toxin function (Zhu, 1992).

### K2 precursor and mature toxin structure predictions support the proposed domain organization and reveal structural similarities with the killer toxin SMK

Next, we generated structure predictions of K2 to be able to explore sequence-structure–function relationships and, in doing so, to further support our proposed organization of the K2 precursor.

**Fig. 5:**
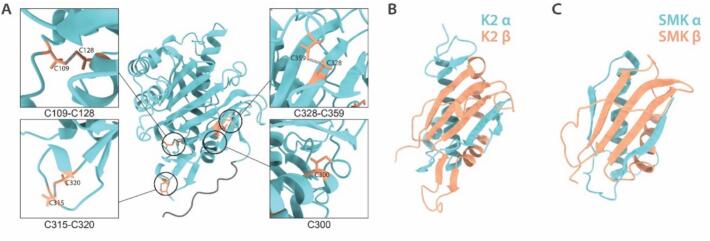
**Protein structure predictions of the K2 precursor and mature K2 toxin.** (**A**) The K2 precursor structure was predicted using AlphaFold. For visibility, the N terminus was truncated up to position 68 (grey). The six essential cysteines form disulfide bridges, whereas the non-essential C300 is not involved in disulfide bridge formation. (**B**) The predicted mature K2 toxin structure. The α subunit (residues 80–164) and β subunit (residues 269–362) form the mature toxin. (**C**) The SMK toxin structure (Protein Data Bank (PDB) ID: 1KVE).

We first predicted the structure of the K2 precursor. This structure folds in the early secretory pathway before Kex2p-dependent processing. The resulting structure (Fig. 5A) showed an average pLDDT score of 79.0 between residues 80 and 362 (containing the proposed α-γ-β domains), while residues 1–79 formed a long, unstructured, rather extended loop with low confidence in the predicted position of residues (pLDDT 19.0) (**Supplementary Table S9**). This was consistent with our proposed K2 precursor organization where this region is a preproregion that is cleaved off.

Further, structural analysis revealed consistency with our alanine-scan-identified critical cysteine residues: three intrasubunit disulfide bridges were present, one within the α subunit (C109-C129) and two within the β subunit (C315-C320 and C328-C359), with residue C300 not involved in a disulfide bridge (Fig. 5A). Furthermore, the proposed Kex2p cleavage sites at positions R79, R165, R221 and R268 were all located within loops and solvent exposed, further substantiating that these sites were indeed accessible cleavage sites for Kex2p.

We subsequently predicted the structure of the mature K2 toxin, using residues 80–164 for the α subunit and residues 269–362 for the β subunit (associated scores: pLDDT 79.8, pTM 0.815, ipTM 0.847) (Fig. 5B, **Supplementary Fig. S7**).

To assess structural differences between the two predictions, we superposed the precursor and mature K2 toxin structure predictions. Overall, the α and β subunits showed very similar structures, however, the α-subunit C-terminal region (residues 153 to 164) exhibited notable differences. Specifically, the RMSD for the β subunit (94 residues) was 2.648 Å, while the α subunit (residues 80–164) had an RMSD of 10.691 Å, which decreased to 2.511 Å when only considering residues 80 to 152.

Next, we compared the predicted mature K2 structure with four available experimental YKT structures: HMK, KP6, KP4, and SMK (PDB IDs: 1WKT, 4GVB, 1KPT, 1KVE, respectively). Our primary aim was to determine whether structural similarities existed between K2 and these other YKTs despite their lack of sequence homology. Interestingly, we found striking structural similarities with the heterodimeric SMK toxin from the halotolerant yeast *Millerozyma farinosa* **(**Fig. 5C**).**

The SMK precursor consists of 222 amino acids and is organized into an N-terminal preregion followed by α-γ-β-subunits (Fig. 4C). The α subunit (residues 19–81) and β subunit (residues 146–222) form the mature heterodimeric toxin (Fig. 5C) (Suzuki and Nikkuni, 1994). The K2 and SMK primary sequences do not share significant sequence identity (**Supplementary Fig. S8**) but share a similar hydropathy profile with a hydrophobic α subunit (**Supplementary Fig. S9**). Further, K2 and SMK both do not contain intersubunit disulfide bridges that link the α and β subunits (Kashiwagi, 1997) – which was observed for the K1 and K28 toxins (Gier, 2019, Suzuki, 2017) (Fig. 4C).

When comparing the structures, the α subunit of K2 and SMK showed a similar fold topology, and both contained a split left-handed βαβ motif, a motif reported to be typically rare in proteins (Kashiwagi, 1997). The α subunit of both toxins contained a central hydrophobic α helix that was largely shielded from the environment by the surrounding β sheet (Fig. 5). Our alanine scan revealed that this central hydrophobic helix (residues 125–148, within sublibrary segments 5 and 6) was critical for K2 toxicity, as only three library variants in this region retained full functionality (**Supplementary Fig. S3**). Further, both SMK and K2 contained a conserved disulfide bridge within the α subunit, formed by residues C37-C53 in SMK and C109-C128 in K2.

The overall fold of both β subunits was also similar, with both containing another rare split left-handed βαβ motif and both containing a conserved stabilizing disulfide bridge at the same location within the structure (C328–C359 in K2, C190–C219 in SMK). Where K2 encoded the nonessential C300 residue, SMK encoded the residue M173. As one major difference, the K2 β subunit featured a larger loop (residues 309–323) containing an additional disulfide bridge (C315–C320), which largely accounted for the difference in size between both toxin’s β subunits (K2: 94 residues; SMK: 77 residues).

In context, next to structural similarities, SMK and K2 also share functional similarities. The SMK toxin has an optimum activity at pH 2.5–4.0 which steeply decreases with increasing pH and is completely lost at pH 6.0 (Suzuki and Nikkuni, 1989). It was suggested that SMK acts by formation of ion channels (Suzuki and Nikkuni, 1989), and later it was demonstrated that it associates with and affects membranes of sensitive cells and induces calcein leakage from liposomes *in vitro* (Suzuki, 2001). Overall, these results pointed to a functional and structural relatedness of the two toxins.

**Fig. 6:**
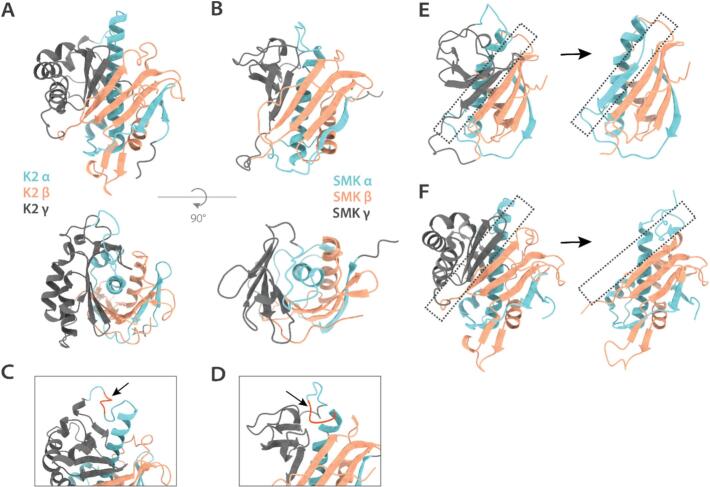
**Comparison of K2 and SMK precursors and mature toxins.** (**A**) The predicted K2 precursor structure is shown, together with the top view (the preproregion was truncated to a few residues). (**B**) The predicted SMK precursor structure is shown, together with the top view (the preregion was truncated to a few residues). (**C**) The GGPGG motif in the K2 precursor loop that connects the α domain to the γ domain is indicated in red. (**D**) The GGGG motif in the SMK precursor loop that connects the α domain to the γ domain is indicated in red. (**E**) In SMK, the β strand of the γ subunit that completes the β sheet is replaced by a β strand of the α subunit after removal of the γ subunit (indicated by the dashed boxes). (**F**) The γ subunit in K2 also donates a β strand to the β sheet, but in the mature K2 toxin predicted structure we only observed formation of a short β strand by the α subunit at the same position.

Next, we predicted the SMK precursor structure (average pLDDT score 81.5) to further explore structural similarities between the two toxins. Both toxin’s precursors showed similar topologies (Fig. 6, Fig. 6).

We specifically used both predicted precursor structures to analyze structural similarities within the γ subunits with the aim to further substantiate its existence in K2. Despite some differences in the γ subunit – the γ subunit of SMK is smaller than the one from K2 (61 versus 101 amino acids, respectively) and the γ subunit of SMK only consisted of β strands, while the one of K2 consisted of both α helices and β strands – they seemed to connect the SMK/K2 α and β subunits in a similar way: First, in both cases, the γ subunit was connected to the α subunit by a loop. The SMK toxin encodes a GGGG motif within this loop region (G72-G75), and the K2 toxin encodes a similar GGPGG motif (G157-G161) within this connecting loop (Fig. 6, Fig. 6, **Supplementary Fig. S8**). Mutation into alanine of several of these residues resulted in reduced toxin functionality (GGPGG had functional scores 3, 2, 3, 4, 3, respectively) (**Supplementary Fig. S3**), suggesting that the region performed a function. We speculate that this motif may provide a required flexibility in the loop connecting the α and γ subunit.

Second, after comparison of the precursor and mature toxin structures, excision of the γ subunit seemed to lead to structural re-arrangements within both SMK and K2. Specifically, within the SMK precursor, the γ subunit provides a β strand to the β sheet that is jointly formed by the α and β subunit (Fig. 6E, left panel). Comparing the SMK precursor structure and the mature SMK toxin structure, it appears that this β strand is − after excision of the y subunit − replaced by a β strand donated by the C terminus of the α subunit (Fig. 6E). The K2 γ subunit behaved in a similar way, it also donated a β strand to the β sheet that was jointly formed by the α and β subunit, but after γ subunit excision, the K2 α-subunit’s C terminus only formed a small replacing β strand while the remainder of the C terminus appeared disordered, pointing into the solution (Fig. 6F).

In summary, beyond confirming structural similarities, we believe that comparing precursor and mature toxin structure predictions provides an opportunity to reconstruct and predict the structural rearrangements that occur during precursor processing into the mature toxin along the secretory pathway.

### Molecular dynamics simulations confirm the overall stability of the predicted mature K2 structure, reveal a more stable α-subunit C-terminal conformation, and support a model of structural rearrangements during processing in the secretory pathway

To confirm that the predicted structure was stable and to assess if the C terminus of the α subunit could possibly find a less disordered, more stable conformation that closer resembles the conformation observed in SMK, we performed molecular dynamics (MD) simulations with the predicted mature K2 toxin structure. As a complementary dataset, we also performed simulations using the SMK toxin. Since this structure was experimentally determined at an acidic pH (pH 3.5), it should be stable in MD simulations (Kashiwagi, 1997).

First, we added missing hydrogen atoms and used the PROPKA algorithm (Olsson, 2011) to assign protonation states to titratable amino acid side chains in SMK and K2. Accurate protonation is crucial, as protonated glutamic and aspartic acids in SMK can form hydrogen bonds that influence pH-dependent toxin stability (Kashiwagi, 1997).

**Fig. 7:**
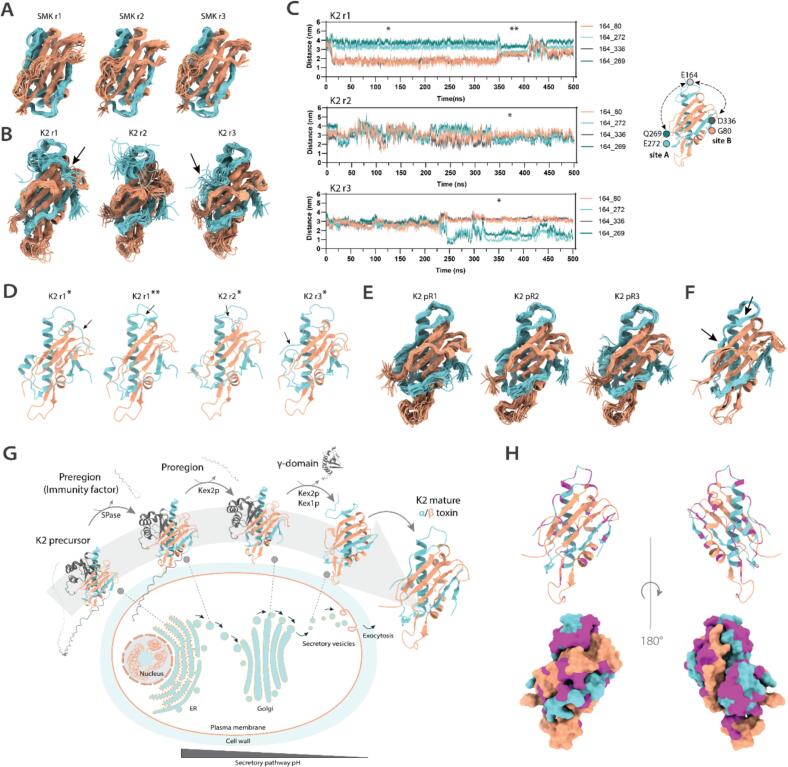
**Molecular dynamics simulations of mature toxin structures.** (**A**) Displayed are 33 superposed frames from each replicate trajectory of the SMK toxin (r1-r3), equidistant in time, between 20–500 ns. (**B**) Displayed are 33 superposed frames from each replicate trajectory of the K2 toxin (r1-r3), equidistant in time, between 20–500 ns. The arrow indicates the position of the C-terminal region of the α subunit. (**C**) The distances between residue 164 and residues 269 or 272 (‘site A’), and 80 or 336 (‘site B’) are shown over time in the three replicates. The timeframes of cluster centroids are indicated by asterisks. (**D**) Cluster centroids from K2 r1-r3 are shown: *, largest cluster, centroid; **, second-largest cluster centroid. The arrow indicates the location of the C-terminal region of the α subunit. (**E**) Displayed are 29 superposed frames from each replicate trajectory of the K2 toxin with altered protonation state (pR1-pR3), equidistant in time, between 20–300 ns. (**F**) The centroids of the largest clusters from pR1-3 were superposed, two fragments of β sheet formation are indicated by arrows. RMSD: 1.155 ± 0.085 Å. (**G**) Overview of K2 processing from the precursor to the mature K2 toxin. Within the ER, the signal peptide (preregion) is cleaved off and forms the immunity factor, protecting the cell against the K2 toxin. Within the Golgi, the Kex2p and Kex1p proteases cleave at several processing sites, removing the proregion and the γ domain. The resulting α and β subunits form the mature secreted K2 toxin, after the C-terminal region of the α subunit adopts a position parallel to the β sheet formed by the β subunit. (**H**) Residues with functional scores equal to or less than 3 and surface accessibility ≥ 25 % visualized on top of the mature K2 toxin structure, colored purple.

Next, we performed three independent 500 ns-long MD simulations of K2 using the expected protonated state at pH 4.0. The radius of gyration (Rg) and number of hydrogen bonds were stable over time, indicating that the structure overall remained folded and compact (**Supplementary Fig. S10**), supporting the accuracy of the predicted structure. For K2, we observed some fluctuation in the Rg and RMSD. Further, the root mean square fluctuation (RMSF) indicated that there were regions with conformational flexibility, particularly the C terminus of the K2 α subunit (**Supplementary Figs. S10A-S10C, S10I and S10J**). Specifically, we inspected the trajectories by displaying 33 superposed frames that were extracted equidistantly in time from each generated trajectory between 20–500 ns (Fig. 7, Fig. 7). These frames created a representation of the sampled conformations over the course of the trajectories and revealed variable behavior of the C-terminal loop of the K2 α subunit across the three replicates: shifting to the right in replicate 1, staying central in replicate 2, and moving to the left in replicate 3 (Fig. 7B, r1–r3).

We also observed these movements when determining the distance between the Cα atom of residue E164 (the C terminus of the α subunit) and the Cα atoms of residue Q269 or Q272 (called herein site ‘A’), or between E164 and residue D336 or G80 (herein site ‘B’) (Fig. 7C**, Supplementary Note 5**). We subsequently performed cluster analysis and the centroids from the largest clusters showed representative structures of these conformations (Fig. 7, Fig. 7). While the position of the α-subunit C terminal region in replicate 3 was more similar to the conformation observed in the SMK toxin toward the end of the trajectory (Fig. 7D**,** Fig. 7A), there was still fluctuation in the position (Fig. 7, Fig. 3, **Supplementary Fig. S10J**). We reasoned that these differences could result from protonation states. In the initial PROPKA prediction, D270 and the C-terminal end of E164 were unprotonated, carrying negative charges during MD simulation. Because E164′s local environment changed with the conformational shift and D270 appeared near the new α-subunit C terminus, their predicted pKa values might no longer be accurate. To potentially enable more stabilizing interactions, we used the centroid structure from replicate 3 (Fig. 7, Fig. 3*) as input for another set of 300-ns triplicate MD simulations, this time protonating D270 and the E164 C terminus. The Rg, RMSD, and number of hydrogen bonds rapidly converged in these trajectories and remained stable throughout the rest of the simulation (**Supplementary Fig. S11**).

We inspected these trajectories further, here displaying 29 superposed frames that were extracted equidistantly in time from each generated trajectory between 20–300 ns (Fig. 7E). These frames show that the α-subunit C-terminal region, located parallel to the β sheet formed by the β subunit, was highly stable over the course of the timeframes. The superposed centroid structures of the main clusters were also highly similar (RMSD: 1.155 ± 0.085 Å) (Fig. 7F). Fragmented β-strand formation was established by backbone interaction between residues Q155 and E281 and between residues G161 and Y276 (Fig. 7F). We observed that the D270 sidechain remained solvent exposed, suggesting its protonation had little effect on structural stability, consistent with its functional score of 4 indicating side-chain mutational tolerance.

This new K2 structure is therefore topologically more similar to SMK than the initial structure prediction. In fact, we speculate that the initial structure prediction in Fig. 5B may represent an early conformation of the α/β toxin after proteolytic excision of the γ domain. After the γ domain is removed, the C terminus of the α subunit adopts the site, which was previously occupied by the γ domain, a process represented by the here obtained MD trajectory (a rearrangement that can occur within ∼ 250 ns (Fig. 7, Fig. 3)).

In summary, these MD simulations helped to refine the K2 structure and allowed speculating on the dynamic structural re-arrangements in the secretory pathway, which we summarize in Fig. 7G.

### Sequence-structure–function analysis reveals functionally important surface-exposed patches and allows mapping of gain-of-function and critical residues

Using the refined mature K2 toxin structure, we analyzed sequence-structure–function relationships, focusing on the functionally important residues and the gain-of-function residues. We had already determined that six of the 26 highly critical residues (functional score 1) within the mature K2 are cysteines, essential due to involvement in disulfide bridge formation (Fig. 5A).

We first examined the overall location of the critical residues by looking at their solvent accessibility: Among the 179 residues of the mature K2 toxin, 91 (51 %) were at least partially solvent exposed (≥25 % solvent accessible surface area of the full residue), with seven of them being highly critical residues (six with score 1, and one with score 1.5) **(Supplementary Table S10 and S11)**. Thus, the majority of critical residues was mostly buried, with only 23 % being ≥ 25 % solvent exposed (six out of 26 residues with score 1). Regarding potential surface interaction sites with target cells, alanine substitutions in interaction sites may only cause a moderate functional loss, since often multiple mutations are required to fully disrupt such a binding site (Gray, 2017). Therefore, we listed all residues with functional scores of 1–3 that were also solvent-exposed (score 1–3, ≥25 % solvent accessible surface area; **Supplementary Table S12**) and mapped them onto the K2 structure. This revealed distinct surface patches which could be further investigated for potential functional relevance, such as involvement in interactions with target cells **(**Fig. 7H**)**.

Next, we mapped each critical residue (functional score 1) with its local contacts, which allows speculation about explanations for the observed loss of function **(Supplementary Fig. S12)**. For example, Y181 and Y212, as well as W273 and Y350, form π interactions likely important for structural stability. Many residues were located in hydrophobic pockets, forming local interactions that alanine substitutions are unlikely to replace (including W85, Y92, F94, Y120, Y132, Y148, W154, W230, L304, I308, and V355, similar to Y211 and Y242 (both with a functional score 2)). Certain residues appeared potentially critical due to steric constraints: mutating G144 to alanine may cause hinder with the nearby E281, while G345 lies adjacent to the C328-C359 disulfide bond with Y276 located right above, constricting the available space. R221 interacts only with D185 on the surface and is most likely critical because of the Kex2p cleavage site.

We further used the computational prediction tool DDmut that predicts mutation-induced effects on protein stability (through changes in Gibbs free energy, ΔΔG) (Zhou, 2023), to explore whether critical residues correlated with predicted effects on K2 stability. Even though low DDmut scores (indicating destabilization) captured most residues with low functional scores from the alanine scan, the overall correlation between functional and DDmut scores was weak and thus rather inconclusive.

We also mapped local contacts for the two gain-of-function mutations that had shown the highest impact **(Supplementary Fig. S13)**. L293 lies in a hydrophobic pocket, while G357 is located in a solvent-exposed C-terminal loop. Why these two positions lead to enhanced function and altered target specificity remains elusive and needs to be further investigated. The solvent accessibility of G357 may contribute to the observed wider mutational tolerance compared to the buried L293 which forms many contacts with surrounding residues (Fig. 3, Fig. 3**, Supplementary Fig. S13**).

### A superfamily of YKTs (SMK, K2, KP4, K1, K66, KHS1, K45) and YKT-like proteins with structural similarities

**Fig. 8:**
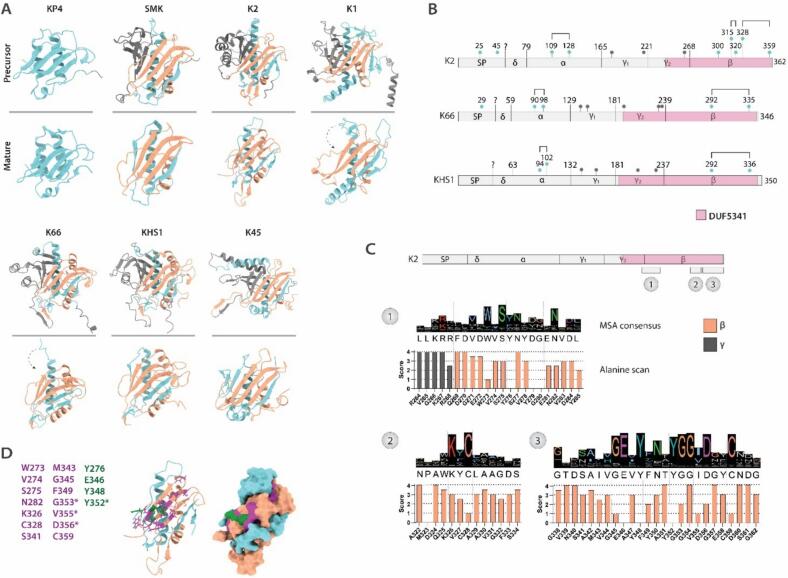
**A superfamily of structurally similar YKTs and DUF5341.** (**A**) Overview of (predicted) precursor structures and mature killer toxin structures. KP4 PDB ID: 1KPT, SMK PDB ID: 1KVE. The mature K2 toxin structure resulted from the MD simulations. For the predicted mature K1 and K66 structures we expect that the α-subunit C terminus was not properly predicted and that there should be an adjustment as indicated by the arrows, similar to what we had observed for K2. See **Supplementary Table S9** for prediction scores and used residues. (**B**) The DUF5341 location is shown in K2, K66 and KHS1 precursors as described on InterPro (IPRO35237). (**C**) Through sequence-based homology searches and multiple sequence alignments (MSA), several conserved residues were identified, especially in three regions within the β subunit. The MSA consensus sequence was compared to data from the alanine scan. (**D**) We list residues that were both conserved and appeared important from the alanine scan (purple), and those that were missing in our sequence-function map but which we expect to be important based on the level of conservation (green). Asterisks mark residues with ≥ 25 % solvent accessible surface area. The patch on the surface indicates residues that may be important in a putative binding site.

We then asked whether more known YKTs could be added to this structural family of K2 and SMK. We therefore manually consolidated a list of sequences with a similar gene organization (using published reports or sequence re-analysis) and predicted their precursor and mature toxin structures based on what we learned from our analysis of SMK and K2 (Fig. 8A**, Supplementary Note 6,** **Supplementary Table S9**). First, we included K66 and KHS1 since these toxins share a DUF5341 domain with K2 (Vepštaitė-Monstavičė, 2018, Rodríguez-Cousiño, 2017), despite low overall sequence identity with K2 (K2-KHS1, K2-K66 or K66-KHS1 share 20.7 %, 18.2 % or 35.5 % sequence identity, respectively). KHS1 is a chromosomally encoded killer toxin in *S. cerevisiae*, which was found to be related to K2 through iterative BLAST searches and it has been speculated that it may be the result of integration of a cDNA copy of the M2 killer virus into the yeast nuclear chromosome (Frank and Wolfe, 2009). Like K2, KHS1 is also heat-sensitive and active against the human pathogen *Nakaseomyces glabrata* (Vijayraghavan, 2023). The K66 toxin is a variant of K21 found in *Saccharomyces paradoxus* and is satellite dsRNA encoded (Vepštaitė-Monstavičė, 2018, Rodríguez-Cousiño, 2017). We further included K1, as the gene organization of the K1 toxin has been well studied (Gier, 2020), is similar to that of K2, and both K1 and K2 target the yeast membrane. We further included K45 based on observed similarities in gene organization compared with K2. Lastly, we included the monomeric KP4 toxin from *Ustilago maydis* (dsRNA virus encoded), which has an experimental 3D structure available that had already been compared to SMK previously, showing that their folding topology was highly similar (Fig. 8A) (Kashiwagi, 1997). Interestingly, all of these toxin structures contained two of the rare left-handed split βαβ-motifs (Kashiwagi, 1997).

There were also notable differences in the heterodimeric structures. For example, the predicted K1 structure contained an inter-subunit disulfide bond between C92-C239, consistent with what was previously determined through cysteine substitutions (Gier, 2019). The predicted structure of K45 contained a disulfide bond at a different location within the β domain than the other toxins (**Supplementary Fig. S14**). Therefore, it may be possible to further divide these toxins into (structural) subfamilies as more toxin sequences and structures become available.

Encouraged by these structural similarities, we next asked whether additional YKT-like proteins existed in yeast genomes and whether combining sequence alignments of YKT and YKT-like proteins with our alanine scanning data could reveal conserved, functionally important residues. While not all of our above investigated toxins contained an annotated DUF5341 domain, we used the DUF5341 region as a search query. The role of the DUF5341 is unclear, but it was pointed out before that this domain is present in numerous proteins of Ascomycota yeasts including several toxins, specifically their β-subunit (K66, KHS1 and K2) (Fig. 8B). Using Interpro, we found a total of 104 entries of DUF5341-containing proteins. Taxonomically, these proteins originated primarily from ascomycete yeasts, with a few sequences derived from (killer) viruses. From this initial set of 104 sequences, we manually curated 34 DUF5341-containing sequences that exhibited a gene organization similar to K2. Similarity was defined based on precursor length, presence of Kex2p cleavage sites, cysteine pattern and the hydropathy profile. Among these 34, four were K2 toxin sequences. We further expanded this list by using the K2 β subunit sequence – which contains a DUF5341 domain – as input for PSI-BLAST iterations to retrieve additional related sequences. This resulted in approximately 100 additional entries. Besides K2, K66 and KHS1 sequences, all of the sequences were uncharacterized proteins. We selected several sequences from different yeast species for precursor structure predictions, revealing structures with a similar topology to K2 **(Supplementary Fig. S15)**. Whether or not this list contains active toxin sequences remains to be shown, but this analysis showed that YKTs are structurally related to many Ascomycete proteins in databases – often proteins with unknown function and a DUF5341.

To identify conserved residues within these YKT and YKT-like proteins we chose 59 representative sequences (identified based on clustering by sequence identity) and performed a multiple sequence alignment. This highlighted, besides the conserved patterns that we used for sequence selection (cleavage sites, cysteines and a hydrophobic region in the α subunit), conserved features within the β subunit (Fig. 8C, **Supplementary File S1)**. Several of these conserved positions coincided with positions that were also associated with loss or diminished function in our alanine scan (functional score 1–3) (Fig. 8C). Residues K326, C328, E346, Y348, G353, G354, D356 and C359 were conserved in over 90 % of sequences, and more positions contained highly conserved amino acid properties (**Supplementary Table S13**). For example, position W273 and Y276 contained either W/Y/F in 94 % and 88 % of the sequences, respectively. We visualized these conserved and functionally important residues on the predicted K2 structure, revealing that several of these residues were also solvent accessible (Fig. 8D). We speculate that these residues within the β subunit (and the DUF5341) may shape an interaction site on the protein surface, for example for the interaction with cell wall β-1,6-glucan or other target structures in Ascomycetes.

## Discussion

While YKTs hold potential as a new source of antimicrobials, a lack of sequence-function and structural data of these often multi-step processed proteins limits engineering strategies to overcome their sometimes inherent limitations. Here, we generated an alanine scanning-based sequence-function map of the K2 toxin from *S. cerevisiae* which revealed key residues for toxin action, gain-of-function variants, and variants with altered target specificity. Using our library data, subsequent genetic analysis and structure predictions, we could reveal and substantiate the existence of previously overlooked non-canonical proteolytic processing sites which led to a proposed novel genetic organization of the K2 ORF. Enhanced by MD simulations, our structure predictions led to a model of how the α-subunit C terminus re-arranges after the excision of the γ domain. Together, these findings provide a detailed sequence-structure–function map that offers a framework for elucidating K2′s molecular mechanisms and guiding its engineering toward enhanced functionality. Moreover, we identified several YKTs with similar predicted folds, suggesting the existence of a structure-based toxin superfamily.

Several points ask for more discussion. **K2 engineerability:** We report several mutations that led to increased zones of inhibition, in particular at positions 293 and 357. A few other reports of naturally occurring killer toxin variants also indicated enhanced functions. A K1 killer toxin variant (BJH001) with the two mutations I103S and T146I increased the cytotoxicity against the yeast *Kazachstania africana* (Crabtree, 2019). A few previous reported K2 variations also influenced the activity spectrum (Zhong, 2024). These data indicate that a small number of mutations can already alter the toxicity to specific species of yeast. While several mutations at positions 293 and 357 resulted in enhanced activity against *S. cerevisiae,* these variants had not been reported in natural isolates yet. Perhaps in nature, toxicity toward an array of yeast species (*i.e.,* wild-type K2 spectrum includes *N. glabratus*) is favored over high toxicity toward the own species (*i.e.,* the L293A variant shows increased activity against *S. cerevisiae*, but little against *N. glabratus*) (**Supplementary Fig. S5**). These data suggest room for engineering of natural occurring variants toward increased activity specifically against pathogenic or spoilage yeast. Alternatively, with more sequenced dsRNA virus genomes more natural variants with altered activity ranges may be detected (Crabtree, 2023). The data presented here show that site-directed mutagenesis libraries can further provide mutants with enhanced functions, even by single-amino acid conversions. We have further shown that an epitope tag can be tolerated at the N terminus of the β domain, providing a tool for future studies.

Further, it is currently unclear how pH-dependent YKTs can maintain a stable conformation within the secretory pathway, where initially pH conditions exist that would render the mature toxin inactive and potentially unstable. The pH difference between endoplasmic reticulum (pH ∼ 7.4) and relatively acidic Golgi (pH ∼ 5.8), toward the secretory vesicles (pH ∼ 5.5) can influence protein stability (Zeng, 2022, Paroutis, 2004). Speculatively, the proregion and γ domain may play a role in stabilizing the structure toward the late Golgi where the pH is sufficiently lowered (Fig. 7G). It was shown that the SMK subunits dissociate at neutral pH (Kashiwagi, 1997, Suzuki and Nikkuni, 1994), where K2 is also unstable (Lukša, 2016), and mature toxins cannot be regenerated (Suzuki, 1997). Such pH-dependent heterodimer stability may be the result of interactions between carboxyl groups of protonated aspartic acid and glutamic acid residues, which act stabilizing under acidic conditions (Kashiwagi, 1997). Further analysis of the generated K2 precursor and mature toxin structures may yield insights into such pH-dependency (e.g. E346 and D356 side chains are in close proximity and seem important for function (Fig. 8D)) and potentially inspire strategies toward engineering pH-optima.

**Attention to non-canonical processing sites for studying YKTs:** We defined cleavage sites at R79 and R165 in the K2 precursor, which had so far been overlooked (Ramírez, 2017). Because killer toxins can use non-canonical Kex2p cleavage sites, but are dependent on a P1 arginine, we found it is a useful approach to mutate the P1 site to determine its essentiality. K1, K28 and K2 (all are toxins encoded on *S. cerevisiae* dsRNA satellites of dsRNA mycoviruses) all use only a moderately processed recognition signal for Kex2p at the proregion cleavage site, indicating there may be a underlying functional relevance selecting for moderate cleavage efficiency (Bader, 2008, Bevan, 1998). In addition, the K74 toxin from *Saccharomyces paradoxus* (encoded on the satellite M74 dsRNA) contains a similar genetic organization and also contains a putative noncanonical proregion ‘VR_36_′ Kex2p cleavage site (Rodriguez-Cousino, N., et al., 2022). An R36K mutation resulted in secreted but inactive K74 heterodimers (Rodriguez-Cousino, N., et al., 2022). It has been speculated that the proregion of these toxin precursors may be involved in inactivation of the toxin until it is secreted (Gier, 2020).

**Protein processing in the secretory pathway:** This study shows, using K2 as a model, that recent developments in high-throughput oligonucleotide synthesis combined with library screening, high-throughput sequencing, protein structure predictions and MD simulations can help to structurally study the multiple steps in processing of secreted proteins. This important and universal process within the secretary pathway was before the structure-prediction age only accessible for very few proteins with structural information available about their precursors or processing intermediates (Shiryaev, 2013, Recacha, 2016).

**Toxin (super)families:** Increasing evidence supports the existence of killer toxin families in yeasts. For example, a family of biologically active K1-like killer toxins has recently been identified (Fredericks, 2021). In this study, we found that both the precursor organization and mature structure of the K2 toxin closely resembled those of the SMK toxin from *M. farinosa* (Suzuki and Nikkuni, 1994, Suzuki and Nikkuni, 1989). Structural comparisons further revealed strong similarities among K2, KP4, K1, K66, K45 and KHS1 toxins **(**Fig. 8**)** (Kashiwagi, 1997, Vepštaitė-Monstavičė, 2018), suggesting that these toxins share a conserved folding topology and domain architecture despite limited sequence identity. All of them contain two left-handed split βαβ-motifs, a rare structural feature, and the majority exhibits comparable precursor organizations with conserved Kex2p cleavage sites, cysteine patterns, and, in several cases, a DUF5341 domain. While these toxins share a common structural framework, they display distinct functional characteristics. For instance, the SMK toxin does not rely on β-1,6-glucans for activity, as *kre1* deletion mutants remain sensitive (Kashiwagi, 1997), whereas K66 interacts with β-1,6-glucans similarly to K2 (Vepštaitė-Monstavičė, 2018). Such functional divergence may reflect evolutionary adaptations.

The β domain, which largely overlaps with the DUF5341 domain, proved particularly valuable for extending our analysis of toxin families. Using it as a guide, we identified a much broader set of structurally related proteins across Ascomycete yeasts, most of which are currently annotated as hypothetical proteins with unknown function. Many of these proteins share the same precursor organization and conserved motifs as known killer toxins, structure predictions revealed strikingly similar folds, and several also contain the DUF5341 domain. This suggests that DUF5341-containing proteins form a wider structural superfamily, which may include both known toxins and yet-undiscovered toxin-like proteins. Even if not all are biologically active toxins, their shared architecture points to a common evolutionary origin and highlights the potential of structure- and domain-based searches to uncover new members of the killer toxin lineage within fungal genomes. Since expression of the α domain of K1 and K2 results in a suicidal phenotype (Prins and Billerbeck, 2024, Prins and Billerbeck, 2025), we envision that heterologous α-domain expression in a model organism like *S. cerevisiae* could provide a useful initial screening method for toxicity of potential novel but functionally similar killer toxins.

## Conclusion

The data presented here describe several novel findings related to the K2 killer toxin and can provide useful information for follow-up site-directed mutagenesis experiments and functional analysis of K2 and predicted toxin family members. Ultimately, a better understanding of relationships between genotype and phenotype of YKTs combined with structural data is needed, together with a better understanding of the molecular processes underlying the killing mechanism.

## Materials and methods

### Strains

For plasmid constructions and maintenance of all constructs *Escherichia coli* DH5α was used (Thermo Scientific, #18265017). Recombinant proteins were expressed in the yeast strain *Saccharomyces cerevisiae* BY4741 (MAT*a leu*2Δ*0 met*15Δ*0 ura*3Δ*0 his*3Δ*1*) (derived from ATCC, 4040002) (Brachmann, 1998). The BY4741 pbs2Δ::KanMX4 deletion mutant with increased sensitivity to the K2 toxin (Servienė, 2012) was obtained from the yeast gene deletion collection (Thermo Scientific, 10277124) and used as a sensitive background strain in halo assays. *Nakaseomyces (Candida) glabratus ySB040* (clinical isolate) was a gift from Dr. Daniel Green (Columbia University Medical center).

### Materials

Media and buffer components were obtained from BD Bioscience (Franklin Lakes, NJ, USA) and Sigma Aldrich (Darmstadt, Germany). Sterile, transparent round-bottom microtiter plates were obtained from Corning (Corning Inc.). Black clear-bottom 96-well microtiter plates were obtained from Thermo Scientific. A Singer ROTOR benchtop robot was used for transfer and re-plating of arrayed yeast cultures and colonies. One-well ROTOR PlusPlates and Singer RePads for robotic transfer were obtained from Singer (SingerInstruments).

### Culture conditions and transformation

*E. coli* cultures were incubated at 37 °C/200 rpm in LB medium, which was supplemented with chloramphenicol (25 µg/mL) or ampicillin (100 µg/mL) were necessary. Solid media were supplemented with 2 % agar and incubated at 37 °C. Preparation and transformation of chemically competent *E. coli* were performed using standard CaCl_2_ and heat-shock procedures.

Before transformation, *S. cerevisiae* cultures were grown at 30 °C on plates containing YPD (1 % yeast extract, 2 % peptone, and 2 % glucose). Transformants were obtained using the LiAc-PEG protocol (Schiestl and Gietz, 1989). Transformants were subsequently selected and cultivated in minimal Synthetic Defined (SD)-AS/Urea media (SD), which unless otherwise mentioned lacked histidine (Prins and Billerbeck, 2021). For selection and maintenance, SD media with 2 % glucose was used as the carbon source. For assay conditions, cells were grown in SD media with 1 % sucrose as the carbon source, 0.5 % galactose was added for induction of protein expression, and media was buffered with 0.5x of McIlvaine buffer at pH 4.6 as described earlier (Prins and Billerbeck, 2021). The K2 toxin is most active in acidic conditions and at a temperature of 25 °C (Lukša, 2016). Cells were either grown on solid media supplemented with 1.6 % agar, in 3 mL liquid media within glass culture tubes, or arrayed in 96-well microtiter plates containing 200 µL of liquid medium. One-well ROTOR plates were filled with 30 mL of solid media.

### Cloning of GFP-dropout entry vectors

All plasmids and primers used within this study are listed in **Supplementary Table S14 and S15**, respectively. To facilitate genetic manipulation, the K2 sequence from the M2 satellite dsRNA was expressed from a pRS423-type high-copy plasmid backbone using the galactose-inducible promoter (pGAL1) from the widely used yeast toolkit (Lee, 2015) (Fig. 1A). In a previous study, the K2 ORF was divided into 16 segments (Fig. 1B) (**Supplementary Table S1**), and each segment was replaced by an SbfI-restriction site, resulting in plasmids pRP035-pRP050 (Prins and Billerbeck, 2024). These plasmids contain a pRS423-type high-copy backbone, and a galactose-inducible promoter. To create the mutagenesis library we used a method based on oligonucleotide pools and Golden Gate cloning (Macia Valero, 2025). First, we generated entry vectors by using the SbfI-sites to insert a Golden-Gate-compatible GFP-dropout module which can be expressed in *E. coli* (Fig. 1C). For this purpose, plasmids pRP035-pRP050 were digested with the SbfI restriction enzyme and treated with alkaline phosphatase (Thermo Scientific, EF0654). A GFP-dropout module, which can be expressed in *E. coli*, was amplified from plasmid pYTK047 from the Yeast ToolKit (Addgene, Kit#1000000061) (Lee, 2015). DNA amplification was performed using 2X Phusion Hot Start II HF PCR Master Mix (Thermo Scientific, F565L). The primers used for this amplification are segment-specific, and add a Type II restriction enzyme (BsaI) site to the GFP-dropout module. DNA purification was performed using the GenElute PCR Clean-Up Kit (Sigma-Aldrich). The amplicons and the digested plasmids (∼50 ng) were assembled using GeneArt Gibson Assembly HiFi Master Mix (Thermo Scientific, A46627) for 1 h at 50 °C, resulting in plasmids where the K2 segments were replaced by a GFP-dropout module (pRP139-pRP154). The reactions were transformed into *E. coli*, green colonies were selected and the plasmids were purified using the GenElute Plasmid Miniprep Kit (Sigma-Aldrich). The plasmid sequences were verified by Sanger Sequencing.

### Oligonucleotide pool design

The complete alanine scanning library was encoded on and ordered as 16 oligonucleotide (oligo) pools (one for each segment) and cloned into the 16 entry vectors using Golden Gate as outlined in Fig. 1**C-F** (**Supplementary Table S14-S16**) (Prins and Billerbeck, 2024, Valero, 2025). Oligonucleotide pool sequences encoding the alanine scanning variants are listed in **Supplementary Table S16**. All positions (including the start codon, excluding the stop codon) were converted to alanine (GCT) (guided by codon usage frequency). Native alanine residues were converted into glycine (GGT). Segment sequences were ordered from Integrated DNA Technologies (IDT) as single-stranded oPools at a 10 pmol/oligo scale, one for each of the sixteen sublibrary segments. In addition, oligonucleotide pools were designed for saturation mutagenesis of positions 293 and 357 (**Supplementary Table S16**). The oligonucleotide length varied from 69 to 119 nucleotides between segments and as such, the pools contain around ∼ 75 % to ∼ 65 % full-length products, respectively, according to the manufacturer’s website.

### Oligonucleotide pool double-stranding

Given the high sequence similarity among oligos within the mutagenesis pools, the pools were only double-stranded instead of amplified to prevent cross-overs leading to undesired products (Valero, 2025). The lyophilized oligo pools were dissolved in milliQ water to a concentration of 1 µM and double-stranded in two 50 µL reactions using 2X Phire Green Hot Start II Master Mix (Thermo Scientific, F126L) and the primers listed in **Supplementary Table S15**. For the reaction, 2.5 µL of each 10 µM primer stock, and 2.5 µL of the 1 µM oligo pool were used and the reactions were subjected to the following parameters on a thermal cycler: (1) 98 °C for 5 s, (2) annealing for 10 s at the appropriate temperature, (3) 72 °C for 10 s, (4) repeat 1–2-3 another cycle, (5) 72 °C for 30 s, for each pool (Fig. 1E). For each oligo pool, the two 50 µL reactions were pooled together and DNA was purified using the GenElute PCR Clean-Up Kit, eluting in 30 µL. The primers also add sequences to the oligonucleotides such that each 5′ and 3′ end contains BsaI-restriction sites that create overhangs compatible with scarless and in-frame ligation into the appropriate entry vector.

### Library assembly

To assemble the mutagenesis libraries, a 25 µL Golden Gate reaction was prepared for each sublibrary which contained 20 ng of the appropriate entry vector, 10 µL of the double-stranded oligo pool, 2.5 µL of the T4 ligase buffer, 1 µL of the T7 DNA Ligase (NEB, M0318S), and 1 µL of the BsaI-HF®v2 restriction enzyme (NEB, R3733S) (Fig. 1F). The reaction mixtures were incubated in a thermal cycler with the following parameters: (1) 42 °C for 2 min, (2) 16 °C for 5 min, (3) repeat steps 1–2 up to 25 cycles, (4) 60 °C for 10 min, (5) 80 °C for 10 min. The 25 µL reactions were each transformed in 125 µL of competent *E. coli* DH5α cells, which were plated on selective media to obtain single colonies. Per segment, green-white screening was used to select white colonies that carried constructs with a successful exchange of the GFP-dropout module for a mutant oligo. All white colonies were counted and scraped together and added into 500 µL of LB media supplemented with ampicillin and glycerol. Part of the cell suspension was inoculated into 3 mL LB supplemented with ampicillin and incubated overnight at 37 °C/200 rpm, while the remainder was stored at −80 °C. From the overnight culture, plasmids were extracted using the GenElute Plasmid Miniprep Kit and eluted in 30 µL.

Library completeness was estimated using the GLUE webserver tool (https://guinevere.otago.ac.nz/STATS/glue.php) (Patrick, 2003). Based on the number of white colonies, all sublibraries were estimated to be complete (assuming a uniform distribution of mutants) (**Supplementary Table S2**). A high completeness was confirmed by next-generation sequencing (NGS) for all sublibraries (99 % complete) except for the sublibrary of segment 5, which was subsequently successfully recloned. Only few mutations with no or low counts at this stage were I228A, Y276A, G280A and Q311A, and, consistently, these variants were also missing later in the screened library in yeast (**Supplementary Fig. S3**).

### Library transformation into yeast and arraying

250 ng of each sublibrary plasmid pool was transformed into *S. cerevisiae* BY4741. The transformation was plated on selective media to obtain single colonies. Individual colonies were picked and arrayed in 200 µL media (SD media with 2 % glucose and 15 % glycerol) in 96-well microtiter plates. The first column of each 96-well plate was dedicated to control strains: Row 1, 3 and 5 contained strains producing wild-type K2, row 2 and 4 contained non-killer strains producing GFP (facilitating visual confirmation of plate orientation), and row 6–8 contained previously created strains with K2 variants that result in varying halo sizes (Fig. 1H). The plates were incubated overnight at 30 °C, the cultures were used as sources for the killer toxin assays, and subsequently stored at −80 °C.

### Killer toxin activity assays

The 96-well arrayed yeast strains were assayed for secretion of active toxin in a halo assay (Prins and Billerbeck, 2025). The liquid cultures from the 96-well plates were pinned onto solid agar plates (SD media with 1 % sucrose, 0.5 % galactose, buffered to pH 4.6) in technical duplicates using the ROTOR robotic pinner (Fig. 1I). After 3 days, halo assay plates were prepared by seeding a sensitive strain (BY4741 pbs2Δ::KanMX4), cultured overnight in the same media composition, in fresh agar plates with the same media composition (∼7.5*10^7^ sensitive cells in 30 mL of media − assuming approximately 2*10^7^ cells per OD_600_ unit per mL). The colonies from each solid source plate were then replicated onto these assay plates and the plates were incubated at 25 °C for 2 days before imaging zones of inhibition using a FUJI imager (Fig. 1J).

### Analysis of zones of inhibition

Active toxin production was identified by the formation of a zone of growth inhibition. To quantitatively compare the activity of the different variants, we used image-processing software originally developed for quantification of colony luminescence – CFQuant, created by Dafni *et al*. (Dafni, E., et al., 2019). However, the software relies on the color gradient of the luminescent halos to help handle image noise, but the zones of inhibition do not display such a gradient. Therefore, a modified version of the software was used (version 1.7, see **Data availability**), which includes extra options to handle image noise based on the position of the colonies and the circular shape of the zones of inhibition. The modified software allowed quantification of areas of the zones of inhibition, and the sizes of colony areas were also recorded (Fig. 1K). To account for plate-to-plate variation, the areas of the zones of inhibition of the three reference wild-type K2 expressing colonies of each plate were averaged, the other areas of zones of inhibition of the respective plate were normalized to the average wild-type K2 halo size on that plate and then averaged between the two technical duplicates.

We set thresholds for strong effects (*i.e.,* no zone of inhibition) to no effects (*i.e.,* wild-type size) of the mutations on toxin activity. We performed a statistical analysis to set the range of wild-type, which meant taking all the three halos of each plate of the reference wild-type K2 producers into consideration, and establishing what we did not consider to significantly deviate from wild-type (100 %) activity. We used this data to estimate the error distribution of wild-type activity and estimated a 99 % confidence interval (CI) on average of 84.5–115.5 % of the wild-type toxin halo size for what represented wild-type activity. We then set 80–120 % of the average wild-type halo area size as conservative lower and upper thresholds for what is considered wild-type activity. Second, we set a upper threshold of 10 % for the variants with the strongest loss-of-function effect, allowing for some biological variation. Two bins were set in between to assess positions with major to moderate effects. The library variants were therefore grouped based on the quantified halo area sizes relative to the wild-type into four bins: Bin 1 (≤10 % of the original halo size), bin 2 (10–40 %), bin 3 (40–80 %) and bin 4 (>80 %) (Fig. 1L). A number of mutants with gain-of-function effects (halo area size > 120 %) were identified and studied separately.

### Plasmid library extraction from yeast

The genotypes and phenotypes were linked using next-generation sequencing. The K2 ORF of 1089 bases was divided into 5 amplicon regions that could each be sequenced with 250 bp paired-end reads (Fig. 1M). Amplicon 1 covered sublibrary segments 1–3, amplicon 2 sublibrary segments 4–6, amplicon 3 sublibrary segments 7–9, amplicon 4 sublibrary segments 10–12, and amplicon 5 sublibrary segments 13–16. In this way, these amplicons targeted the site of mutagenesis only, such that these regions could be sequenced to high coverage depth. All yeast colonies belonging to the same bin were pooled together per sublibrary segment into 300 µL of 2 % glucose SD media supplemented with 20 % glycerol, in such a way that approximately similar amounts of biomass of each colony were added. Of each cell suspension, 100 µL was subsequently inoculated into 3 mL of SD medium with 2 % glucose and grown overnight at 30 °C, 200 rpm, while the remainder was stored at −80 °C. From these overnights, the OD_600_ was determined, and the cultures belonging to the same amplicons and bins were subsequently inoculated together into 20 mL of fresh media as a first multiplexing step, such that the added inoculum was normalized for the number of colonies that the sample represented. This 20 mL culture was again incubated overnight at 30 °C, 200 rpm in SD medium containing 2 % glucose as the carbon source. The next day, 10 mL of the cultures were centrifuged (2500x *g*, 5 min) and the supernatant was aspirated. For each plasmid extraction, the cell pellet was resuspended in 500 µL of the resuspension buffer of the GenElute Plasmid Miniprep Kit. The samples were kept on ice while the cells were lysed using acid-washed glass beads, with bead beating performed in two cycles at 6 m/s for 20 s (Savant Bio 101 FastPrep FP120 bead mill). 300 µL of the suspension was used for a plasmid extraction following the Plasmid Miniprep Kit manufacturer’s instructions and plasmid DNA was eluted in 30 µL elution buffer.

### Illumina library samples preparation

For primer barcode design, first a set of 407 barcodes of 8 bp was selected (available at https://hannonlab.cshl.edu/nxCode/nxCode/Ready_made_sets.html), and an optimized set of 10 barcode sequences was selected using BARCOSEL (https://ekhidna2.biocenter.helsinki.fi/barcosel/ (Somervuo, 2018), with a minimal pairwise Levenshtein sequence distance of 4. PCR-mediated partial Illumina adapter and 8 bp-barcode addition and library amplification were performed using 1 µL of the purified plasmid library as a template in a 50 µL reaction using the 2X Phusion Hot Start II HF PCR Master Mix, with the following parameters for the thermal cycler: (1) 98 °C for 30 s, (2) 98 °C for 10 s, (3) 56 °C for 20 s, (4) 72 °C for 1 min, (5) 2–3-4 for 20 cycles, (6) 72 °C for 5 min (Fig. 1M). The samples were purified using the GenElute PCR Clean-Up Kit and eluted in 20 µL elution buffer. Fluorometric quantification of the amplicons was performed with the Quant-iT PicoGreen dsDNA Assay Kit (Invitrogen) in a Synergy Mx plate reader (BioTek). For the fluorometric quantification, 1 µL of amplicons was diluted in 99 µL of 1x TE buffer (10 mM Tris-HCl, 1 mM EDTA, pH 7.5) and mixed with 100 µL of the PicoGreen agent (diluted 1:200 in 1x TE) in a black transparent-bottom 96-well microtiter plate. Standards were prepared following the manufacturer’s instructions. Based on these measurements, the amplicons were further multiplexed into five final sequencing samples. The amounts of amplicons were added proportionally to the plate colony numbers in order to maintain an equimolar representation, with the aim of maintaining equal read distribution for all colonies in the downstream sequencing run as much as feasible.

### Sequencing

The samples were sequenced by Genewiz (Azenta Life Sciences, Leipzig, Germany) using the Amplicon-EZ service (250 bp paired-end reads) on an Illumina Miseq sequencing platform (Fig. 1N). Independent validation of a set of individual colony sequences was performed by Sanger sequencing (Macrogen).

### Bioinformatics analyses of sequencing reads

Illumina sequencing returned two FASTQ files containing the forward and reverse sequencing reads and quality information. The initial quality profiles of the reads were visualized with FastQC (v0.11.9 (Andrews, 2010). Sequences were preprocessed in several steps (Fig. 1O), where default parameters were used unless specified otherwise. Paired-end reads were demultiplexed using the 5′ 8 bp-barcodes on forward and reverse reads and assigned to the original segment/bin combinations (allowing one mismatch; –barcode-mm 1) (AdapterRemoval v2.3.3 (Schubert, 2016), and for downstream analysis this barcode was also trimmed at this step. Reads with poor quality were removed and low-quality bases were trimmed from the paired end reads (Trimmomatic (Bolger, 2014) using SLIDINGWINDOW:4:20, MINLEN:100). The paired-end reads were subsequently merged into a single read (ea-utils fastq-join, v1.3.1 (Aronesty, 2013). The quality of merged reads was assessed with FastQC. The reads were mapped to the reference template of pRP002 using BWA-MEM (v0.7.17 (Li, 2013) – of the reads that passed the previous filters, all were mapped − and the alignment files were sorted using SamTools (v1.18 (Danecek, 2021). SamTools indicated good mapping quality (MAPQ alignment score 60). The resulting files were subsequently parsed by a custom python script.

Since the sequencing read covers the entire mutated region, the read from this amplicon is by design representative and sufficient for variant calling and counting. The sequencing results were analyzed to obtain the occurrences of each amino acid conversion. Because of the need to analyze each read as a whole instead of single nucleotide substitutions, we developed a custom script for analysis of the alignment file. The custom script parses through the alignment file and performs stringent quality filtering to retain high-quality reads and assigns counts to the detected variants in the context of the entire read. An alanine mutation is counted when the programmed substitution is present and any additional amino acid variations are absent throughout the entire read. The results are aggregated and merged into excel files for further analysis.

The sequencing yielded a total of 305.651 paired-end reads. The reads were assigned to the respective bin/segment combination by demultiplexing based on the dual barcode sequences. After quality filtering, paired-end reads merging and mapping, 243.321 reads (79.6 %) were yielded for input into a custom script for computational analysis (Fig. 1O). The custom script performed further stringent filtering to yield only high-quality sequencing reads and subsequently performed variant calling and counting of occurrences of single-amino acid mutations. This yielded a total of 224.328 read sequences that passed filtering (92.2 % of input reads), of which 180.222 reads contained single, intended mutations (80.3 % of used reads). The resulting coverage per variant is displayed in **Supplementary Fig. S2A**.

### Functional score calculation

Quantitative data of read counts were normalized for differences in the total read counts of the five different sequencing samples. Overrepresented outliers were determined by applying a mathematical criterion for outliers (the third quartile (Q3) + 1.5 times the interquartile range (IQR)). Subsequently, for each amino acid position in the K2 ORF, the read counts across bin 1–4 were compared. We set the following thresholds for determining the functional score: 1) a minimum threshold for the coverage of ten variant read counts, together with either 2) a minimum of 80 % of all variant reads of that position detected within one bin, or 3) a minimum of 80 % of all variant reads of that position was present in two adjacent bins. If criteria 1) and 2) were met, we assigned a functional score that is equal to the bin number (*i.e.* a variant detected with > 80 % of the reads in bin 3 was assigned a functional score of 3). If criteria 1) and 3) were met but not criterium 2), we assigned a functional score of the average of the two adjacent bins (*i.e.* if a variant was detected in bin 3 and 4 it was assigned the score 3.5). If criterium 1) was not met, we excluded the reads from further analysis and the position was assigned a score of 0. As such, the variants obtained a functional score between 1 and 4 with intervals of 0.5 points, or score 0.

### Construction of double mutant L293A + G357A

A construct containing both L293A and G357A mutations was generated by amplification of the respective regions from the two isolated plasmids pRP120 and pRP121 using primers RP01 and RPA25 (**Supplementary Table S14 and S15**). The pRP002 plasmid backbone was digested with EcoRI (Fast Digest, Thermo Scientific, FD0274) and BamHI (Fast Digest, Thermo Scientific, FD0054), and the PCR products were digested with either EcoRI and ApaLI (NEB, R0507) (containing the L293A mutation) or ApaLI and BamHI (containing the G357A mutation). All products were purified from an agarose gel using the GenElute Gel Extraction Kit (Sigma-Aldrich) and ligated in a 20 µL ligation reaction using T4 ligase. After transformation into *E. coli* and subsequent plasmid purification from a monoclonal culture, the sequence was verified by Sanger sequencing.

### Manual halo assay

To assess secretion of active toxin, separate variant colonies were picked and inoculated in 3 mL of SD media (2 % sucrose, 1 % galactose, pH 4.6) and incubated at 25 °C at 200 rpm for 16 h. A sensitive strain (*S. cerevisiae* BY4741 pbs2Δ::KanMX4 (Servienė, 2012) was also included. The OD_600_ of all overnight cultures was determined and adjusted to 10. Of these cell suspensions, 5 µL (∼10^6^ cells) was spotted onto an agar plate seeded with 125 µL of the OD_600_ 10 culture per 10 mL of solid media (same media composition) of the sensitive strain (∼2.5·10^7^ cells). Plates were incubated at 25 °C for 2 days before imaging. ImageJ (v1.52a) was used for quantification of these halo sizes. For enhanced visibility, the brightness and contrast of the images was adjusted equally.

### Spot assay

To assess suicidal phenotypes (Prins and Billerbeck, 2025), separate variant colonies of the strains of interest were picked and inoculated in 3 mL of SD media (2 % sucrose, pH 4.6) and incubated at 25 °C at 200 rpm for 16 h. The OD_600_ of all overnight cultures was determined and adjusted to 10. In a transparent round-bottom 96-wells microtiter plate, a 10-fold dilution series from the OD_600_ 10 culture was prepared in sterile water. From each dilution, 5 µL was spotted onto two target plates: The first one containing 2 % glucose as a carbon source (repressing condition), the second buffered to pH 4.6 and containing 2 % sucrose as a carbon source and supplemented with 1 % galactose (inducing condition). The plates were incubated at 30 °C or 25 °C, respectively, for 3 days before imaging the growth. For enhanced visibility, the brightness and contrast of the images was adjusted equally.

### Protein structure predictions

Using AlphaFold (v2.3.1) (Evans, 2021, Jumper, 2021) and ColabFold (v.1.5.2) (Mirdita, 2022) (installed locally), several protein structure predictions of monomers or multimers were generated. ColabFold was run with increased recycle counts (up to 50) and seeds (up to 100) (using models alphafold2_ptm or alphafold2_multimer_v3). Models were ranked by pLDDT, pTM and ipTM scores. Top ranked structures were relaxed using the AMBER colaboratory from ColabFold with default settings (relax_amber.ipynb) (Mirdita, 2022). UCSF ChimeraX (v1.6.1) and Pymol were used for analysis and visualization of the predicted protein structures.

### Molecular dynamics simulations

All simulations were prepared using GROMACS (v2023.1) with the CHARMM36m all-atom force field (Huang, 2017, Abraham, 2015). Since the pKa of titratable residues can shift depending on the local environment, pKa values were estimated using PROPKA (v.3.5.1) (Olsson, 2011), with default parameters. The protonation state of the proteins was adjusted to reflect conditions at pH 4.0.

The proteins were placed into dodecahedral boxes, which a minimal distance of 1.0 nm between the protein and the box boundaries (Bondi, 1964). For the K2 system, the box dimensions were 3.62 x 5.26 x 4.31 nm, and for SMK, they were 4.18 x 3.33 x 3.77 nm. The systems were solvated with TIP3P waters (K2: 11.780 molecules; SMK: 5.699 molecules). To neutralize the system and achieve a 0.15 M NaCl concentration, a corresponding number of water molecules was replaced with Na^+^ and Cl^-^ ions.

All simulations used parameters recommended for the CHARMM force field. The leap-frog integrator was used with a 2 fs time step. Neighbor searching utilized the Verlet cutoff scheme, and van der Waals (Lennard-Jones) interactions were smoothly switched off between 1.0 to 1.2 nm. Coulomb interactions were computed using the Particle-Mesh Ewald (PME) method, with a real-space cutoff of 1.2 nm and a grid spacing of 0.16 nm (Hub, 2014). The V-rescale thermostat maintained the temperature at 298 K with a time constant of 0.1 ps, while the Parrinello-Rahman barostat maintained pressure at 1 bar using isotropic scaling and a time constant of 2 ps (Bussi, 2007, Parrinello and Rahman, 1981). Bond lengths involving hydrogen atoms were constrained with the LINCS algorithm (Hess, 2008).

After system construction, the potential energy of the system was minimized using the steepest descent method. This was followed by 100 ps of position-restrained equilibration in the NVT ensemble and 100 ps of equilibration in the NPT ensemble. Once temperature and pressure equilibration were achieved, unrestrained production runs of 500 ns were generated (298 K, 1 bar) with a 2 fs time step, and trajectory data were recorded every 10 ps. Trajectory analysis was conducted using GROMACS built-in tools, with hydrogen bonds defined by a distance cutoff of 3.5 Å and an angular cutoff of 30°.

### Multiple sequence alignment

Sequences containing the DUF5341 domain were collected from InterPro (#IPRO35237). The 104 resulting sequences were manually filtered based on precursor length, the presence and pattern of potential Kex2p recognition sites and cysteine residues, and the hydropathy profile (TMHMM2.0 and DeepTMHMM) (Hallgren, 2022, Krogh, 2001). In addition, homology searches using the K2 β subunit (which largely overlaps with the DUF5341 domain) were performed with PSI-BLAST to identify additional related sequences, subjected to the same filtering step (Altschul, 1997). The resulting list of sequences was clustered using mmseqs2 based on 80 % sequence identity (https://toolkit.tuebingen.mpg.de/tools/mmseqs2). The multiple sequence alignment was performed using Clustal Omega with default parameters (Sievers and Higgins, 2018).

For two open reading frames, we had indications based on sequence comparison to K2/K66/KHS1 that the automated exon annotation was likely incorrect. We therefore re-analyzed the annotation in the raw genomic data and used corrected versions of the ORFs: A0AAN7WN76 (edited version “eA0AAN7WN76”) and CAI5026128.1 (edited version “eCAI5026128.1”) (**Supplementary Note 7**).

## CRediT authorship contribution statement

**Rianne C. Prins:** Writing – review & editing, Writing – original draft, Visualization, Validation, Methodology, Investigation, Formal analysis, Data curation, Conceptualization. **Tycho Marinus:** Methodology. **Eyal Dafni:** Methodology. **Iftach Yacoby:** Methodology. **Sonja Billerbeck:** Writing – review & editing, Supervision, Project administration, Funding acquisition, Conceptualization.

## Funding

This work was financially supported by an FSE Research grant from the Faculty of Science and Engineering, University of Groningen (SB), the NWO XS award OCENW.XS3.069 (SB), the Israel Science Foundation 941/22, MOST Grant 4563 and The Council for Higher Education (VATAT) Excellence center - Energy.

## Declaration of competing interest

The authors declare that they have no known competing financial interests or personal relationships that could have appeared to influence the work reported in this paper.

## Data Availability

Data will be made available on request.
