# Supplementary material for: Alanine scanning of the yeast killer toxin K2 reveals key residues for activity, gain-of-function variants, and supports prediction of precursor processing and 3D structure
Source: J Struct Biol X. 2025 Dec 18;13:100142. doi: 10.1016/j.yjsbx.2025.100142 (PMC13404059; doi:10.1016/j.yjsbx.2025.100142)
Supplement: Supplementary Data 2 [file mmc2.pdf]

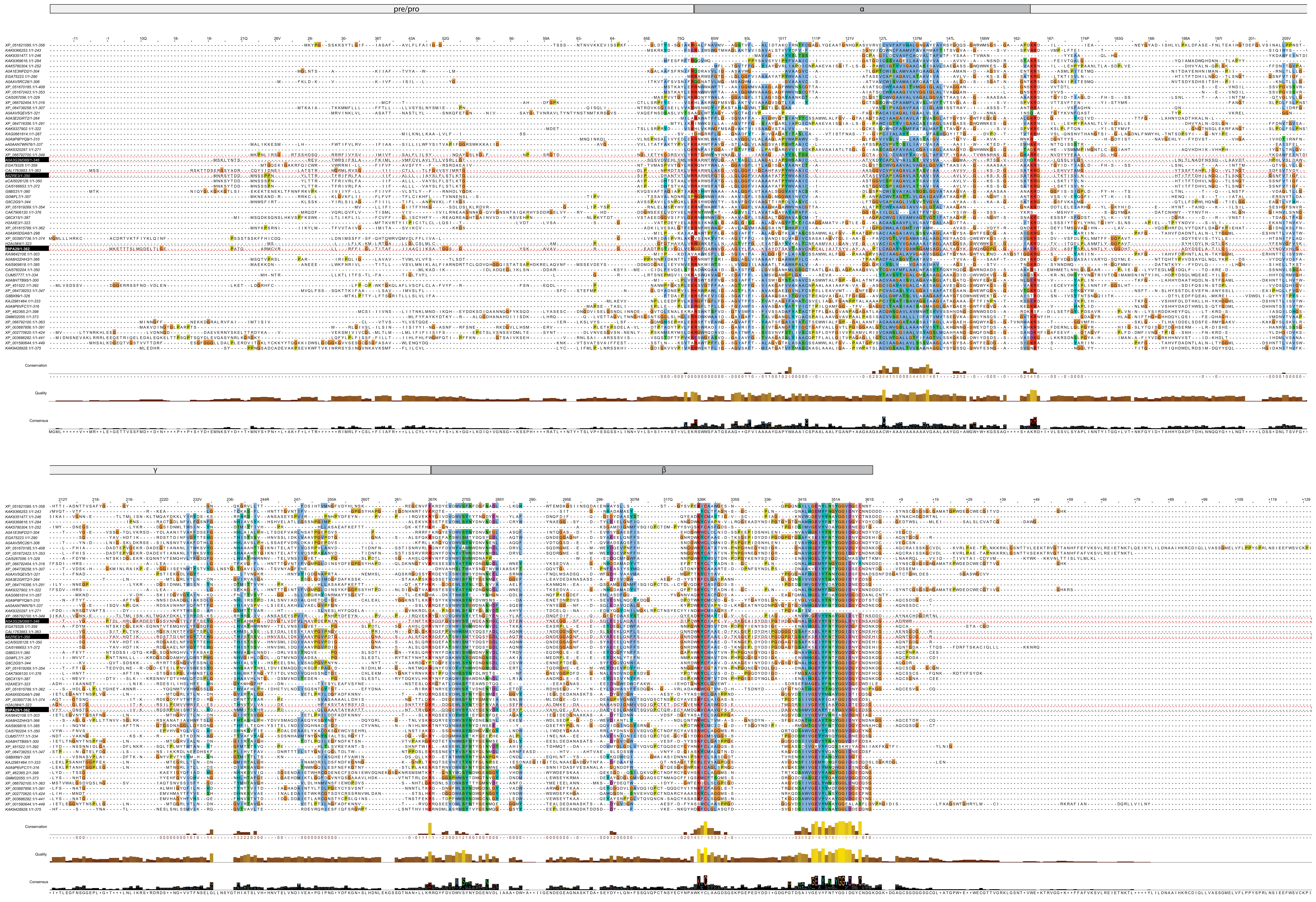

pre/pro
α
γ
β
Conservation
Quality
Consensus
